# Synthetic Routes to Approved Drugs Containing a Spirocycle †

**DOI:** 10.3390/molecules28104209

**Published:** 2023-05-20

**Authors:** Nazar Moshnenko, Alexander Kazantsev, Evgeny Chupakhin, Olga Bakulina, Dmitry Dar’in

**Affiliations:** 1 Institute of Chemistry, Saint Petersburg State University, 199034 Saint Petersburg, Russia; funfunzheza@gmail.com (N.M.); alekaz.2018@gmail.com (A.K.); o.bakulina@spbu.ru (O.B.); 2 Institute of Living Systems, Immanuel Kant Baltic Federal University, 236016 Kaliningrad, Russia; chupakhinevgen@gmail.com; 3 Saint Petersburg Research Institute of Phthisiopulmonology, 191036 Saint Petersburg, Russia

**Keywords:** spirocycles, three-dimensional, high-F_sp3_, spirocyclizations, cyclodehydration, ketalization, intramolecular claisen condensation, intramolecular alkylation, approved drugs

## Abstract

The use of spirocycles in drug discovery and medicinal chemistry has been booming in the last two decades. This has clearly translated into the landscape of approved drugs. Among two dozen clinically used medicines containing a spirocycle, 50% have been approved in the 21st century. The present review focuses on the notable synthetic routes to such drugs invented in industry and academia, and is intended to serve as a useful reference source of synthetic as well as general drug information for researchers engaging in the design of new spirocyclic scaffolds for medicinal use or embarking upon analog syntheses inspired by the existing approved drugs.

## 1. Introduction

Spirocycles are becoming increasingly popular in drug discovery. Inherently three-dimensional, these high-F_sp3_ (F_sp3_ = number of sp^3^ hybridized carbons/total carbon count) motifs are expected to rid drug candidate molecules of many liabilities which would be inevitably encountered with the formerly typical scaffolds built on flat aromatic carbo- and heterocycles. Hence it is perhaps unsurprising that the trend (articulated for the first time in the *Escape from the Flatland* publication by Lovering and colleagues [1]) has now translated itself in an increasing number of approved drugs containing spirocyclic motifs. We and these colleagues recently published a review article on the use of spirocycles in drug discovery [2]. In this review, we partly touched upon the syntheses of approved spirocyclic drugs. However, that overview was far from exhaustive. Moreover, it only episodically touched upon approved spirocyclic drugs, also focusing on advanced, though not approved, drug candidates. Considering the apparent interest in the subject from the research community (at the time of writing this review, publication [2] was cited over 100 times in the 1.5 years since its appearance in the literature), we thought it prudent to compile a more comprehensive compendium of synthetic routes to approved drugs containing a spirocyclic motif, with a particular emphasis on alternative synthetic approaches to the same drug molecule, if such exist. The present review is the result of realizing that intent. The review covers 23 drug molecules approved for medical use over the period of 1959 to 2021.

## 2. Griseofulvin

**Figure 1:**
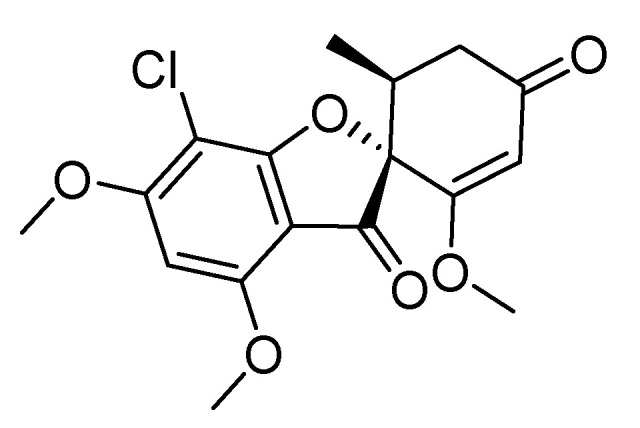
Structural formula of griseofulvin.

Griseofulvin (Figure 1) is an antifungal agent used to treat fungal infections of the fingernails and toes. It is one of the earliest spirocyclic drugs, and was approved in 1959. The exact mechanism of its action remains unclear, with putative targets including tubulin beta chain and keratin, type I cytoskeletal 12 through which griseofulvin interferes with fungal mitosis.

**Scheme 1:**
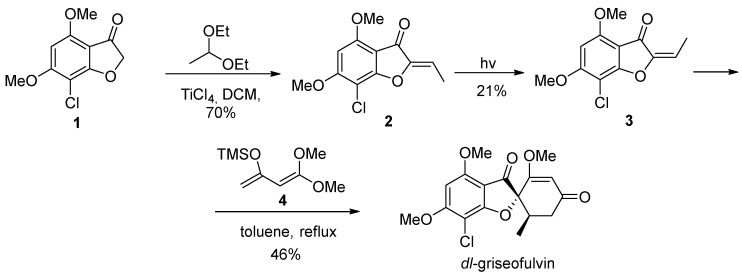
Synthesis of dl-griseofulvin by Yamoto and co-workers [3].

Two total syntheses of griseofulvin were accomplished in the 1990s. Yamoto and co-workers reported the synthesis of racemic griseofulfin in 1990 [3]. The synthesis commenced with 7-chloro-4,6-dimethoxy-3(2*H*)-benzofuranone (**1**) which was condensed with acetaldehyde diethyl acetal in the presence of Ti(IV) chloride to give (*Z*)-configured ethylidene derivative **2**. In order to bring the olefin configuration to the desired (*E*), compound **2** was subjected to somewhat low-yielding photochemical isomerization. The (*E*)-configured olefin **3** was now set for a Diels–Alder reaction with diene **4** (10 equiv.), which was accomplished in refluxing toluene to furnish racemic griseofulvin in 46% yield (Scheme 1).

**Scheme 2:**
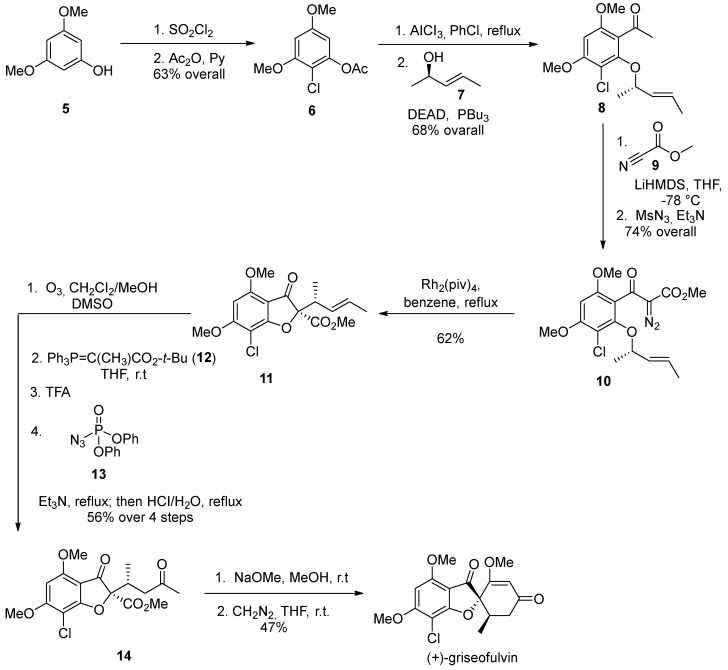
Synthesis of (+)-griseofulvin by Pirrung and co-workers [4].

Pirrung and co-workers reported the first asymmetric synthesis of natural enantiomer (+)-griseofulvin in 1991 [4]. Commercially available phenol **5** was regiospecifically chlorinated to provide intermediate **6**. The latter underwent a Fries rearrangement in the presence of aluminum chloride and the liberated phenol was involved in the Mitsunobu coupling with non-racemic allylic alcohol **7** to give rise to pentasubstituted benzene building block **8**. The acetyl group in the latter was deprotonated and methoxycarbonylated by treatment with Mander’s reagent (**9**) and the resulting β-keto ester was subjected to Regitz diazo transfer to provide the diazo compound **10**. Decomposition of the latter was accomplished with the use of rhodium pivalate catalyst and the resulting rhodium carbene underwent intramolecular trapping with the nearby oxygen atom, followed by sigmatropic rearrangement to give benzofuranone **11**. Conversion of the latter to non-racemic methyl ketone **14** was accomplished via ozonolysis, the Wittig reaction with phosphonium ylide **12**, *tert*-butyl ester cleavage with TFA and decarboxylation on treatment with diphenylphosphoryl azide (**13**), followed by exposure to refluxing aqueous hydrochloric acid. Finally, methyl ketone **14** was subjected to treatment with sodium methoxide on which it underwent the Dieckmann cyclization. The resulting cyclohexane-1,3-dione (griseofulvinic acid) was *O*-methylated with diazomethane to furnish (+)-griseofulvin (Scheme 2). 

## 3. Spironolactone

**Figure 2:**
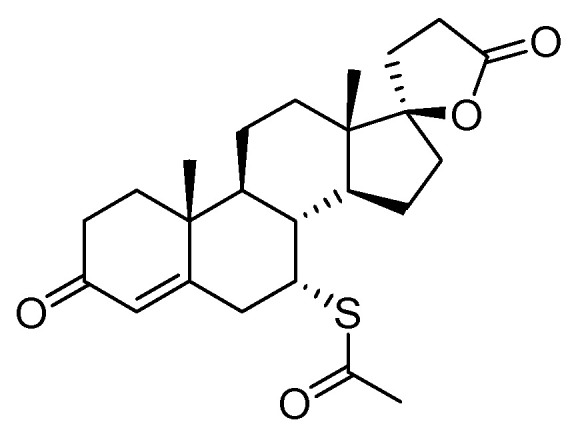
Structural formula of spironolactone.

Spironolactone (Figure 2) is a relatively old multi-target drug that is primarily used to treat high blood pressure and heart failure. Additional therapeutic applications of this drug include edema, cirrhosis of the liver and primary aldosteronism. The mechanism of action of spironolactone includes binding to intracellular mineralocorticoid receptors (MRs) in kidney epithelial cells, which leads to the inhibition of aldosterone binding [5]. 

The first industrial synthesis of spironolactone was reported in 1957–1959 by Cella and co-workers of G. D. Searle and Co. [6,7,8]. The synthesis commenced with previously reported ethynyl steroid building block **15**. It was carbonylated at the alkyne terminus by treatment with methyl magnesium bromide, followed by quenching with carbon dioxide. Hydrogenation of alkyne **16** over the Lindlar catalyst elaborated the propionic acid side chain in **17** required for the formation of the spirocyclic lactone at the next step. Cyclization on treatment with *p*-toluenesulfonic acid yielded unsaturated lactone which was hydrogenated over palladium on carbon to yield spirocyclic lactone **18**. 

**Scheme 3:**
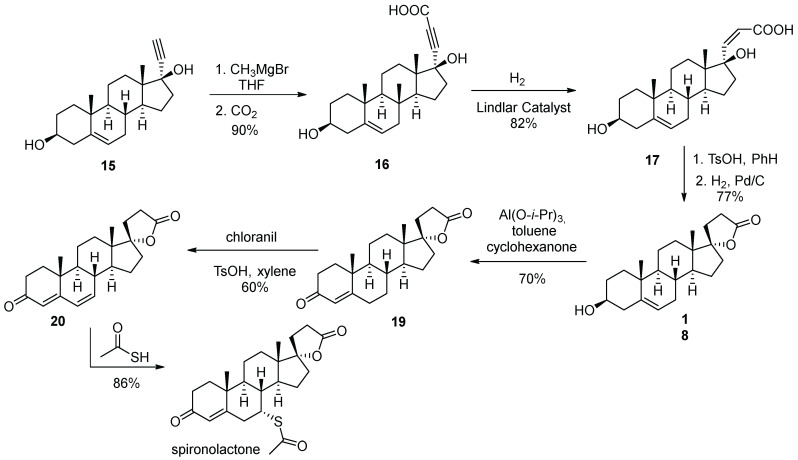
Spironolactone synthesis by G. D. Searle and Co [6,7,8].

Oppenauer oxidation of the alcohol functionality in **18** was accompanied by the migration of the double bond to give α,β-unsaturated ketone **19**. The conjugated system in **19** was extended by γ,δ-oxidation with chloranil to give compound **20**, and set the scene for the conjugate addition of thioacetic acid at the d-position, which proceeded with high diastereoselectivity and furnished spironolactone in 86% yield (Scheme 3). 

**Scheme 4:**
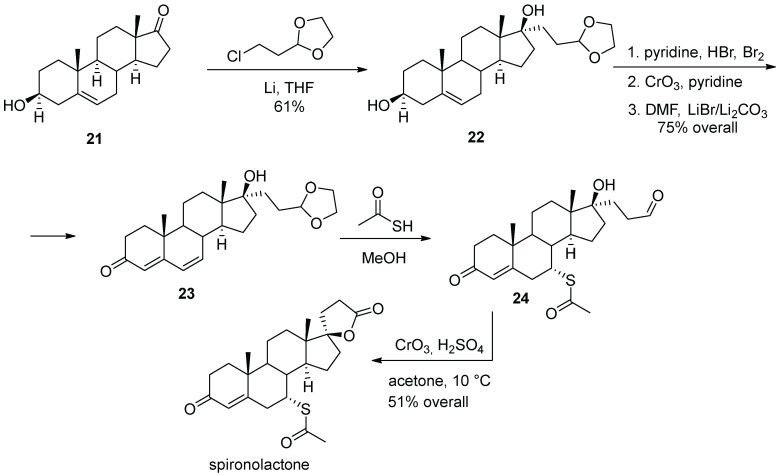
Ciba Geigy industrial synthesis of spironolactone [9].

Another synthetic approach of spironolactone was patented in 1980 by Ciba Geigy scientists [9]. The synthesis stems from dehydroepiandrosterone (**21**), which was treated with 3-chloropropionaldehyde ethylene acetal and lithium to furnish tertiary alcohol **22** in diastereoselective fashion. The following sequence of steps (which proceeded in 75% overall yield) included bromination of the double bond, oxidation of the secondary alcohol to ketone and, finally, base-promoted double dehydrobromination, which led to α,β,γ,δ-unsaturated ketone (androstadienone) **23**. Interestingly, the conjugate addition of thioacetic acid was in this case performed prior to the elaboration of the spirocyclic lactone in the last step. Thioacetic acid also caused the deprotection of the aldehyde to furnish **24**. Oxidation of the aldehyde to the carboxylic acid in the presence of sulfuric acid brought about the closure of the lactone ring and led to the formation of spironolactone in 51% yield from **23** (Scheme 4). 

**Scheme 5:**
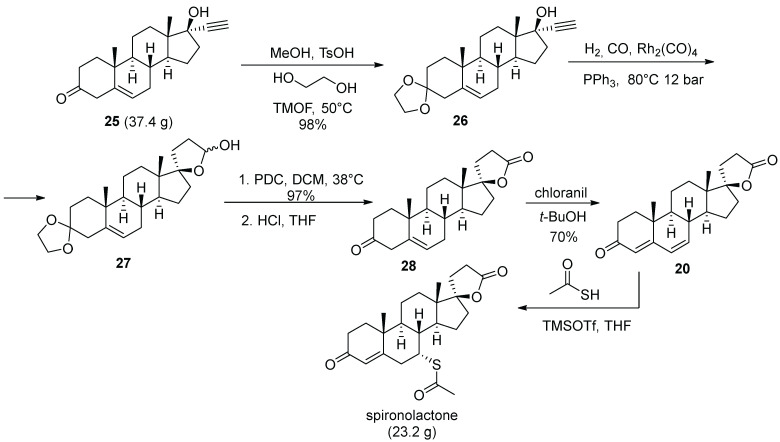
The Upjohn Co. synthesis of spironolactone [10].

A fundamentally different route to spironolactone performed in multigram scale was developed by the Chemical Process Research and Development scientists at The Upjohn Co. in the late 1980s [10]. The synthesis relied on the readily available ethynyl steroid building block **25**, the ketone group of which was protected as ethylene glycol ketal in the first step. The key transformation which led to the formation of spirocyclic lactol intermediate **27** was rhodium-catalyzed hydroformylation at elevated temperature and pressure. Oxidation of **27** to lactone was achieved with PDC and the ketal protecting group was removed to afford **28**. The formation of α,β,γ,δ-unsaturated ketone **20** was achieved with chloranil (vide supra). and conjugate addition of thioacetic acid (catalyzed by TMSOTf) at the δ-position furnished spironolactone (Scheme 5).

**Scheme 6:**
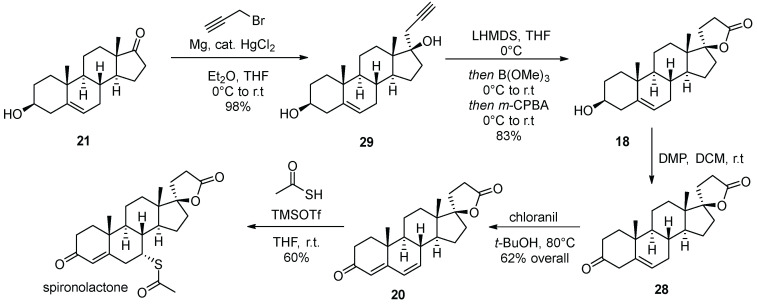
Synthesis of spironolactone via γ-lactonization of homopropargyl alcohol.

Among many non-industrial syntheses of spironolactone described in the literature [5], the most recent one reported by Nagamitsu, Ohtawa and co-workers is quite notable [11]. The synthesis commences with dehydroepiandrosterone (**21**) and is reliant on the innovative approach to γ-lactonization of homopropargyl alcohols, which involves terminal borylation of a homopropargyl moiety followed by *m*-CPBA oxidation. The latter sequence leads to the transformation of the alkyne moiety into a ketene intermediate which is trapped intramolecularly to give a γ-lactone. Hence, the starting material **21** was treated with propargyl magnesium bromide (under Hg(II) catalysis) to give homopropargyl alcohol **29** in nearly quantitative yield. Application of the γ-lactonization protocol described above led to the formation of spirocyclic lactone **18**. Oxidation of the secondary alcohol moiety in the latter with Dess–Martin periodinane (DMP) gave γ,δ-unsaturated ketone **28**. Transformation of the latter into spironolactone was analogous to the Upjohn Co. synthesis described above. Oxidation with chloranil installed the polyunsaturated ketone system (**20**) which is suitable for the conjugate addition of thioacetic acid (also TMSOTf-catalyzed in this case), resulting in the formation of spironolactone (Scheme 6).

## 4. Fluspirilene

**Figure 3:**
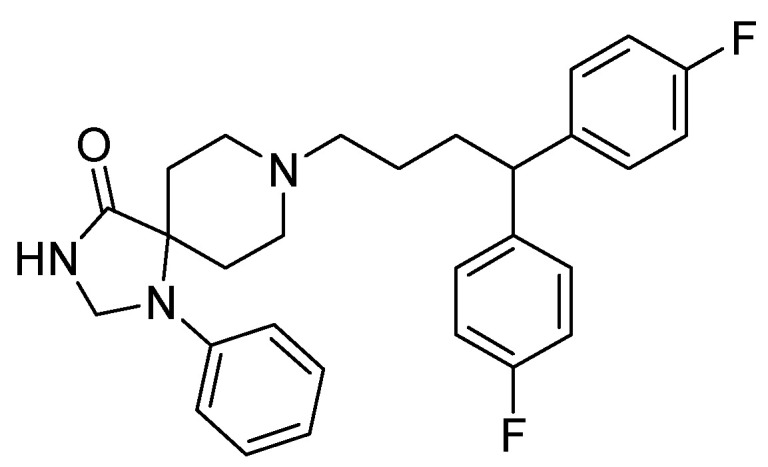
Structural formula of fluspirilene.

Fluspirilene (Figure 3), a spirocyclic antipsychotic drug was discovered at Janssen Pharmaceutica in 1963, and by 1970 it had been approved for the treatment of schizophrenia [12]. The primary molecular targets of fluspirilene in the central nervous system are dopamine D_2_ and serotonin 5HT_2A_ receptors, as well as α1 subunit of voltage-dependent calcium channel [13]. 

**Scheme 7:**
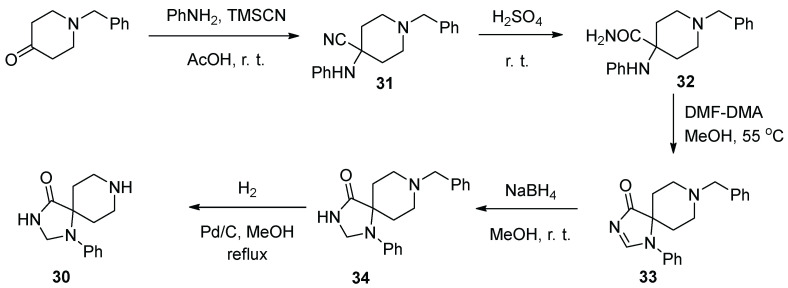
A 2018 synthesis of the core piperidine spirocycle needed for fluspirilene assembly.

Fluspirilene is an old drug, and reviewing the vast patent literature on its synthesis would be tedious. Instead, we will focus on the most notable cases of fluspirilene assembly from the relatively recent literature. For instance, an efficient synthesis of the key spirocyclic building block **30** needed for the synthesis of the drug was published in 2018 by a team of Italian and Polish scientists [14]. The synthesis commenced with 1-benzyl piperidin-4-one, which was involved in the Strecker reaction with aniline and trimethylsilyl cyanide to give nitrile **31**. The latter was hydrated in sulfuric acid and the resulting α-anilino carboxamide **32** was condensed with *N,N*-dimethylformamide dimethyl acetal to form spirocyclic imidazolinone **33**. The C-N double bond in **33** was reduced to give imidazolidinone **34** and the benzyl group was removed by hydrogenation to give the target spirocycle **30** (Scheme 7).

**Scheme 8:**
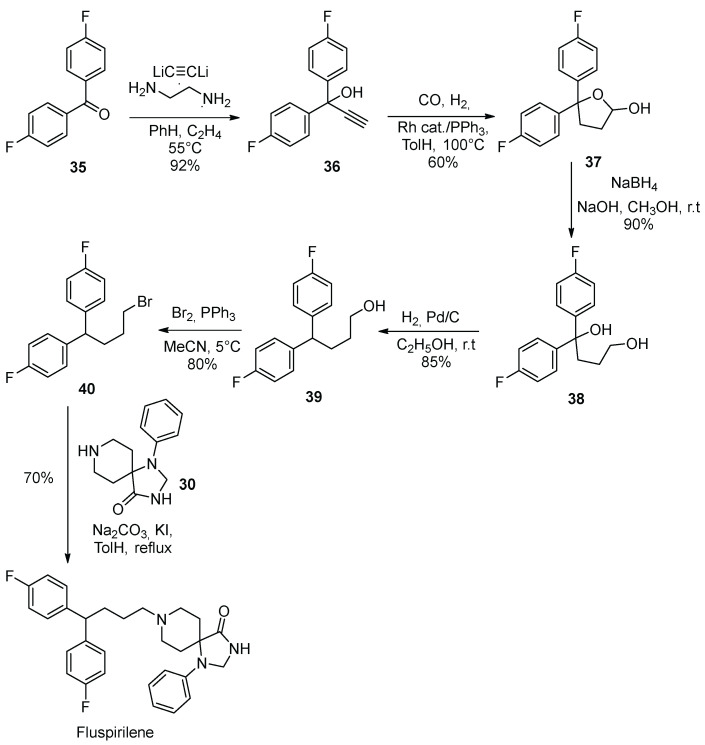
A 2001 synthesis of fluspirilene from an Italian group.

Interestingly, it is the synthetic assembly of the 4,4-bis(4-fluorophenyl)butyl halide building block (for the alkylation of the key spirocycle **30**) that received much of the synthetic route development. For instance, in 2001, an Italian group reported an interesting synthesis of fluspirilene, starting from 4,4′-difluorobenzophenone (**35**). Acetylene bis-lithium salt was added to the keto group of **35** in the presence of ethylene diamine to give allylic alcohol **36**. The latter was hydroformylated in the presence of a rhodium catalysts cyclic hemiacetal **37**. Sodium borohydride reduction of the latter furnished diol **38**, which lost its doubly benzylic tertiary alcohol hydroxy group on hydrogenation. Alcohol **39** was brominated to alkyl halide **40**, which then was used in the completion of fluspirilene synthesis (Scheme 8) [15].

**Scheme 9:**
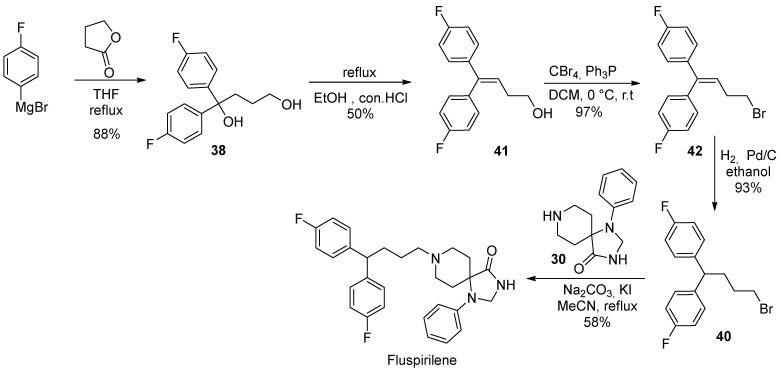
Fluspirilene synthesis by the Shanghai Institute of Organic Chemistry [16].

A different approach to bromide **40** was reported in 2011 by a Shanghai Institute of Organic Chemistry group [16]. Diol **38** was obtained by reacting γ-butyrolactone with excess 4-fluorophenyl magnesium bromide. Thereupon, it was dehydrated in refluxing aqueous hydrochloric acid to give olefinic alcohol **41**, the bromination of which gave unsaturated alkyl bromide **42**. Hydrogenation of the latter resulted in double bond saturation and went on without a loss of the bromide to give alkylating agent **40**, which was used in the preparation of fluspirilene (Scheme 9).

## 5. Fenspiride

**Figure 4:**
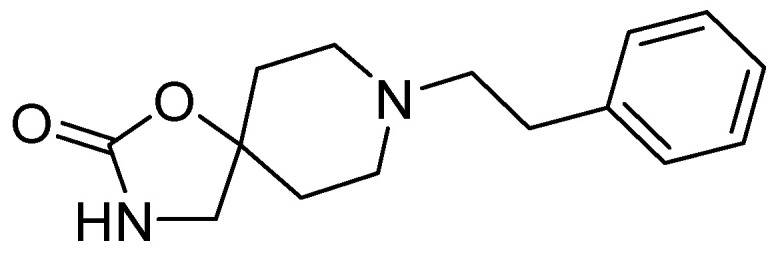
Structural formula of fenspiride.

Fenspiride (Figure 4), a spirocyclic oxazolidinone, acting as an antagonist of a_1_-adrenergic receptors, was first approved in selected European countries for medical use in the 1970s for the treatment of otolaryngological or ENT (ear, nose and throat) organ diseases, as well as respiratory tract diseases [17]. The structure of fenspiride comprises a well-established feature for binding to the hERG channel (an aromatic ring at a certain distance from a basic amine center) [18]. Unfortunately, it does bind to this off target, and causes prolongation of the QT interval. For this reason, i.e. the increased risk of torsades de pointes, fenspiride was withdrawn from the pharmaceutical market in most markets around the world [19].

**Scheme 10:**
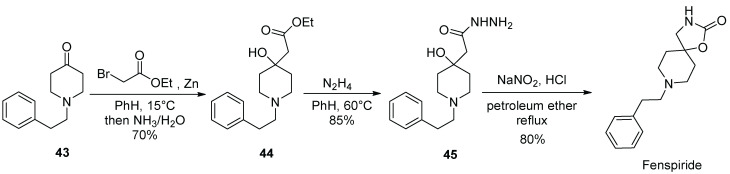
Synthesis of fenspiride by the Voghera company [20].

One of the early patents, obtained by the Italian Voghera company for the synthesis of fenspiride, describes the assembly of the target molecule starting from 1-phenethyl piperidin-4-one (**43**), which was involved in the Reformatsky reaction with ethyl bromoacetate. After the conversion of the resulting β-hydroxy ester **44** to hydrazide **45**, the latter was diazotized, which triggered a Curtius rearrangement of the intermediate acyl azide, and the isocyanate product of the rearrangement was trapped, intramolecularly, by the nearby hydroxy group, to give fenspiride (Scheme 10) [20].

**Scheme 11:**
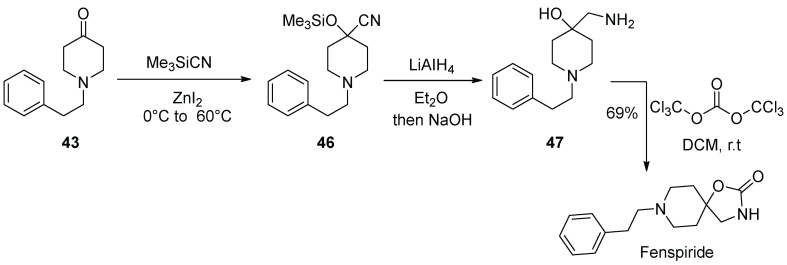
Synthesis of fenspiride by a Mexican group.

In 1994, a Mexican group disclosed an alternative approach to elaborating the spirocyclic oxazolidinone based on 1-phenethyl piperidin-4-one, which includes the Strecker-type reaction of the ketone with trimethylsilyl cyanide. The resulting *O*-silylated a-hydroxy nitrile **46** was subjected to reduction with lithium alumohydride to give β-amino alcohol **47**. The latter was treated with trichloromethyl carbonate, which donated the carbonyl group and resulted in the closure of the oxazolidinone ring of fenspiride (Scheme 11) [21]. 

**Scheme 12:**
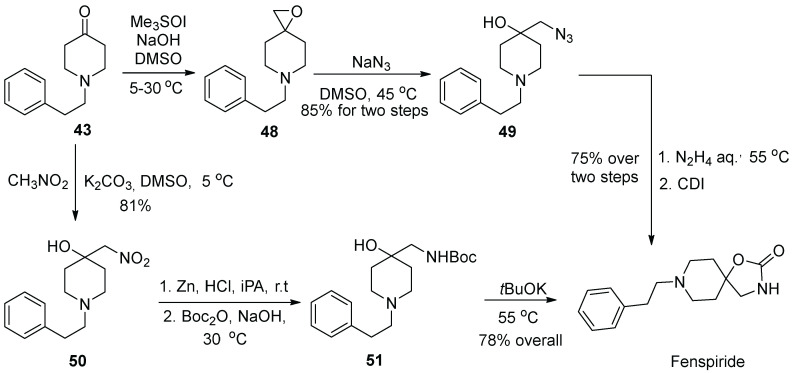
Synthesis of fenspiride by Emcure Pharmaceuticals [22].

In 2019, the Indian company Emcure Pharmaceuticals jointly with a University of Pune group published a completely novel route to fenspiride which is based on the nitro aldol reaction and is amenable to industrial-scale production of the drug substance [22]. In fact, the researchers disclosed two possible approaches to fenspiride for comparison, and reached a conclusion that the nitro aldol route was preferred from the standpoint of pharmaceutical production. In the first approach, Corey–Chaykovsky epoxidation gave epoxide **48**, which was opened up by sodium azide to give β-azido alcohol **49**. Reduction by hydrazine (presumably to alcohol **47**) and treatment with CDI (the donor of the carbonyl) afforded fenspiride. In the second, preferred, approach, ketone **43** was condensed with nitromethane anion and the resulting nitro compound **50** was reduced to the respective amine and Boc-protected to afford intermediate **51**. Deprotonation of the hydroxy group in the latter with potassium *tert*-butoxide triggered intramolecular displacement of the *tert*-butoxy group and ring closure to fenspiride (Scheme 12). Additionally, the second route was performed on a large scale, starting with 1 kg of compound **50** to furnish 880 g of fenspiride.

It is worth noting the effective procedure for the late-stage [^11^C], [^13^C] and [^14^C] carbon isotope labeling of cyclic carbamates recently developed by Audusio and co-workers [23]. The method allows the incorporation of labeled carbon dioxide into azidoalcohol 10 to furnish C-labeled fenspiride in a direct, cost-effective and sustainable manner.

## 6. Amcinonide

**Figure 5:**
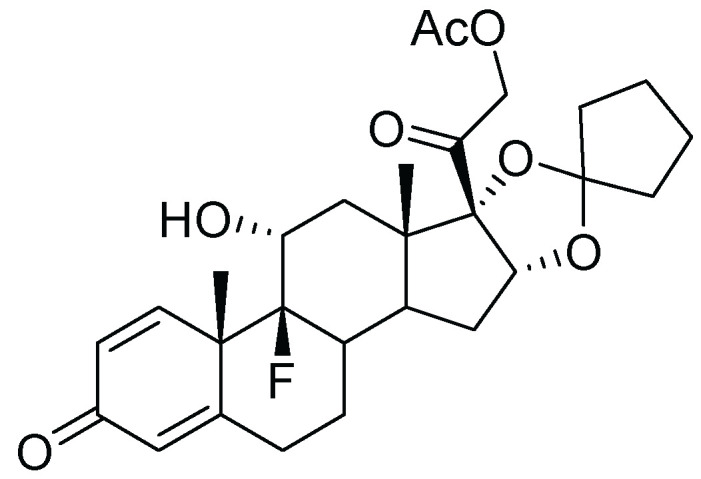
Structural formula of amcinonide.

Amcinonide (Figure 5) is a topical corticosteroid approved in 1979 for the treatment of various inflammatory dermatological conditions such as atopic dermatitis and allergic contact dermatitis. Amcinonide displayed high affinity to the glucocorticoid receptor which is thought to be the main biological target mediating its anti-inflammatory action. Binding to the receptor and its activation by amcinonide eventually leads to the induction of phospholipase A2 inhibitory proteins, which suppresses the production of arachidonic acid and the downstream production of prostaglandins as chemical mediators of inflammation [24]. 

**Scheme 13:**
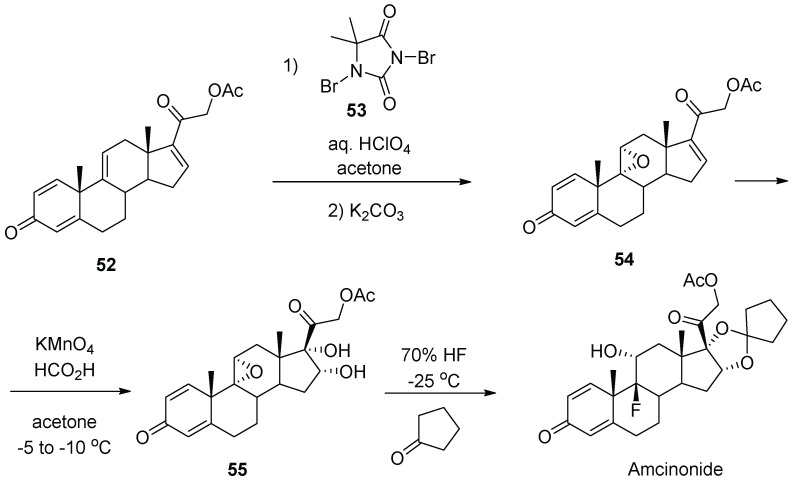
Synthesis of amcinonide.

Unfortunately, synthetic routes to amcinonide are poorly described in the literature. Even the original patent issued to the American Cyanamide company [25] features little synthetic detail. An industrial synthesis of amcinonide patented by an Indian company in 2018 [26] is described in sufficient detail. It starts with readily available natural steroid 2-((10*S*,13*S*,14*S*)-10,13-dimethyl-3-oxo-6,7,8,10,12,13,14,15-octahydro-3*H*-cyclopenta[a]phenanthren-17-yl)-2-oxoethyl acetate (**52**). The latter is subjected to epoxidation of the non-conjugated double bond using 1,3-dibromo-5,5-dimethylhydantoin (dibromantin, **53**) and aqueous perchloric acid, followed by the treatment of the resulting bromohydrin with potassium carbonate. Epoxide **54** is formed with high diastereoselectivity. The next step is dihydroxylation of the cyclopentene ring, which is brought about by potassium permanganate and formic acid in acetone. Finally, diol **55** is formed diastereoselectively, then subjected to the treatment with 70% hydrofluoric acid, followed by the addition of cyclopentanone. The net result of the latter step is epoxide opening with the fluoride anion and spirocyclic ketal formation leading to amcinonide (Scheme 13).

## 7. Guanadrel

**Figure 6:**
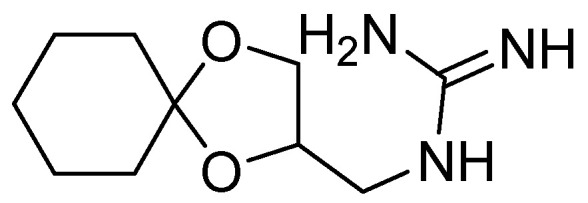
Structural formula of guanadrel.

**Scheme 14:**
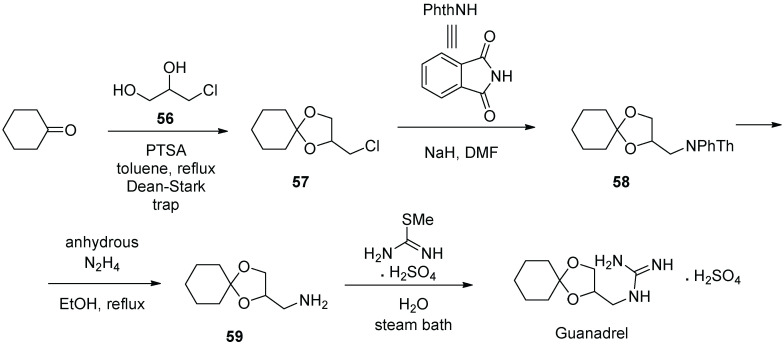
Industrial synthesis of guanadrel.

Guanadrel (Figure 6) is an anti-hypertension drug containing a guanidine moiety which was approved in 1979 [27]. It exerts its therapeutic action by blocking the sodium-dependent noradrenaline transporter, and thus is a typical adrenoblocker [28]. The industrial synthesis of guanadrel was patented in 1970 by its inventors [29]. In the first step, cyclohexanone undergoes ketalization with 3-chloro-1,2-propandiol (**56**), forming 2-chloromethyl-1,4-dioxyspiro[4,5]decane (**57**). The latter building block is used to alkylate phthalimide sodium salt to give the protected amine derivative **58**. Removal of the phthalimide protecting group with hydrazine liberates the primary amino group in **59**, which is reacted with *S*-methyl thiourea sulfate in aqueous medium on steam bath heating to give guanadrel as a sulfate salt (Scheme 14).

**Scheme 15:**
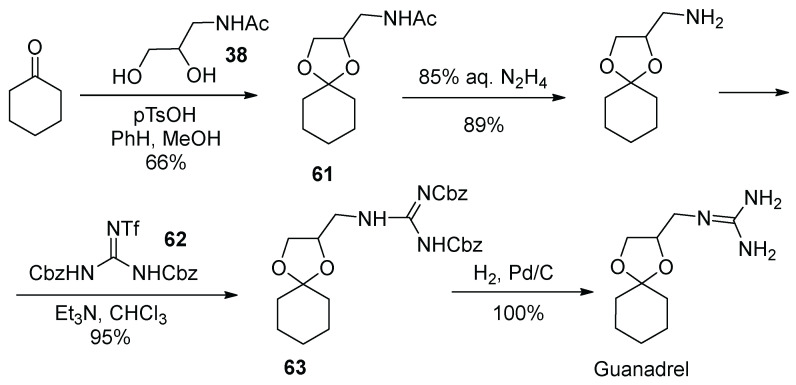
Synthesis of guanadrel by Goodman [30].

In 1999, Goodman of UC San Diego developed a novel guanidine synthesis using triflyl-diurethane protected guanidines and applied it to the synthesis of guanadrel. In this synthesis, cyclohexanone was ketalized with acetylamino-substituted diol **60**. The resulting spirocyclic building block **61** was deacetylated with hydrazine and reacted with *N,N*′-di-Cbz-*N*″-triflylguanidine (**62**). Upon the displacement of the triflic amide, which is a leaving group in **62**, bis-Cbz-protected guanidine **63** was hydrogenated over palladium on charcoal to give guanadrel in quantitative yield as a free base (Scheme 15). Interestingly, the attempted use of Boc protecting groups in lieu of Cbz failed, since the deprotection under acidic conditions led also to the removal of the acid-prone ketal [30].

## 8. Buspirone

**Figure 7:**
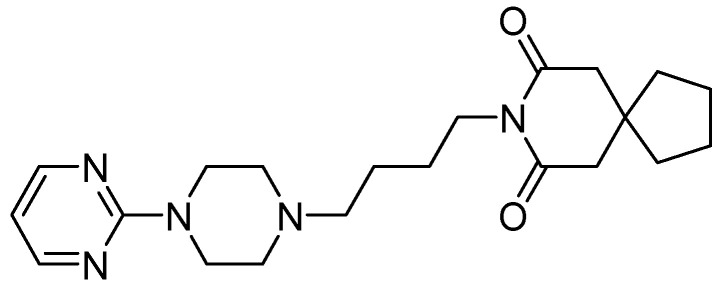
Structural formula of buspirone.

First synthesized in 1966 and approved for medical use in 1986 buspirone (Figure 7), a spirocyclic serotonin 5-HT_1A_ receptor agonist is primarily used to treat anxiety disorders [31]. Several patented protocols for the preparation of buspirone [32,33,34], including industrial synthesis, have been reported in the literature.

**Scheme 16:**
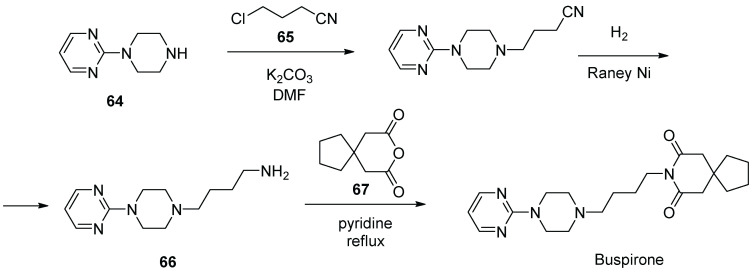
Industrial synthesis of buspirone.

On the plant scale, the following route was initially employed for the production of buspirone. 1-(Pyrimidin-2-yl)piperazine (**64**) was alkylated with 4-chlorobutyronitrile (**65**), and the nitrile group was hydrogenated over Raney nickel catalyst to give butylamine **66**. The latter was condensed with commercially available 3,3-tetramethylene glutaric anhydride (**67**) in refluxing pyridine to give, after crystallization, buspirone [33] (Scheme 16).

Over more than three decades following the approval of buspirone for medical use, several newer synthetic routes appeared in the literature journals, some of which will be discussed below.

**Scheme 17:**
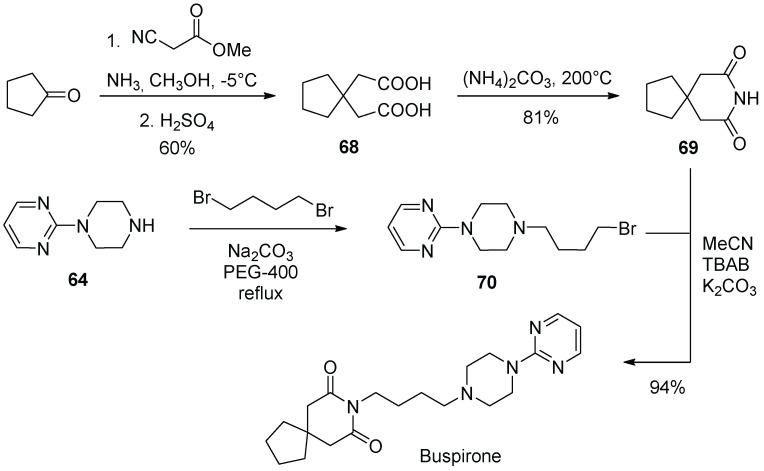
Synthesis of buspirone by Wei and co-workers (2008) [35].

In 2008, Wei and co-workers from China University of Mining and Technology reported a new process for the production of buspirone. In the first step, condensation of cyclopentanone with methyl isocyanoacetate catalyzed by ammonia followed by acidic ester hydrolysis afforded cyclopentane-1,1-diacetic acid (**68**). Heating of the latter with ammonium carbonate at 200 °C afforded 3,3-tetramethyleneglutarimide (**69**). The latter was alkylated with butyl bromide **70** which was prepared, in turn, from 1-(pyrimidin-2-yl)piperazine (**64**) by alkylation with 1,4-dibromobutane. The last step afforded buspirone in nearly quantitative yield (Scheme 17) [35].

**Scheme 18:**
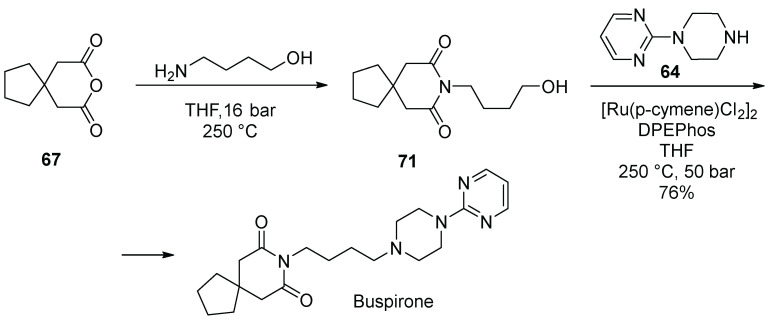
Preparation of buspirone involving piperazine alkylation via hydrogen borrowing.

In 2019, Steven Ley and co-workers reported a continuous flow method to prepare buspirone which involves the direct alkylation of 1-(pyrimidin-2-yl)piperazine (**64**) with alcohol **71** via a Ru(II)-catalyzed hydrogen-borrowing approach (Scheme 18) [36].

**Scheme 19:**
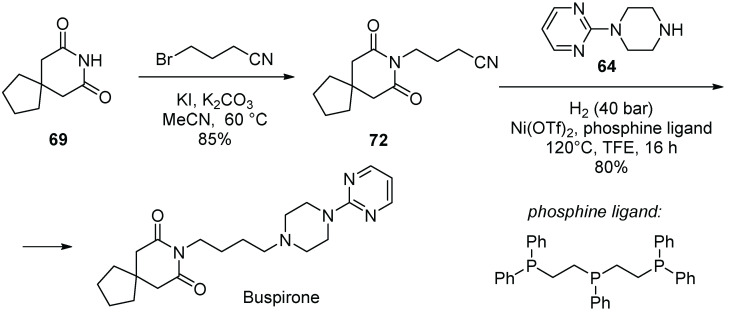
Preparation of buspirone involving reductive cross-coupling of **64** with nitrile **72**.

In 2022, Beller and co-workers reported a remarkable, Ni-catalyzed reductive cross-coupling of nitriles with primary and secondary amines. In order to exemplify the scope of their methodology, a novel synthesis of buspirone was presented. Nitrile **72** prepared by alkylation of 3,3-tetramethylene glutarimide (**69**) with 4-bromobutyronitrile reacted 1-(pyrimidin-2-yl)piperazine (**64**) under 40 bar of hydrogen in the presence of nickel triflate and a special triphosphine ligand to give 80% yield of buspirone (Scheme 19) [37].

## 9. Ivermectin

**Figure 8:**
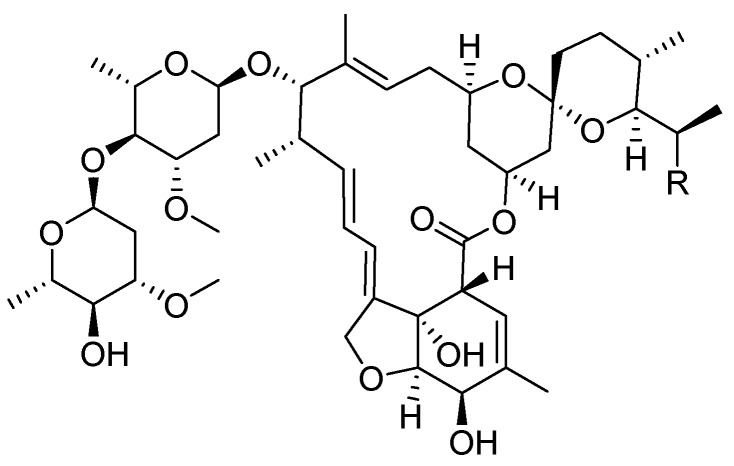
Structural formula of ivermectin.

Discovered in 1975 ivermectin (Figure 8), an antiparasitic drug was initially approved for veterinary use in 1981. After extensive clinical research, ivermectin was approved in 1987 for use in humans, for the treatment for a wide range of parasitic infections [38,39]. Ivermectin acts by binding to parasites’ glutamate-gated sodium channels and forcing them to remain open, which causes the overflow of chlorine anions and, consequently, hyperpolarization of the cell membranes. This eventually kills the parasite [40].

**Scheme 20:**
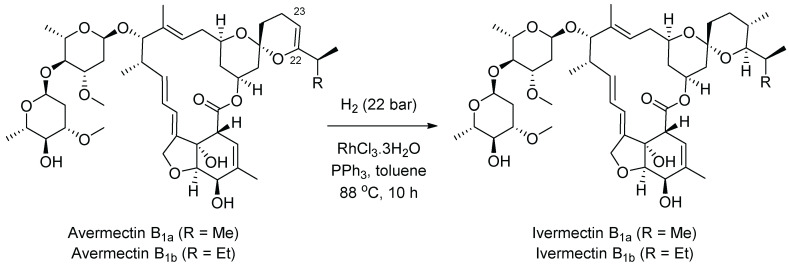
Hydrogenation of avermectins B_1a_ and B_1b_.

Ivermectin is an approximately 80:20 mixture of two individual macrocyclic lactone (macrolide) compounds, ivermectin B_1a_ and B_1b_. These compounds are produced by hydrogenation of the C^22^ = C^23^ double bond of the mixture of avermectins B_1a_ and B_1b_ which are, in turn, produced by fermentation of *Streptomyces avermitilis*, the bacteria discovered in 1970 by Satoshi Ōmura, for which discovery he and William Campbell of Merck were awarded the Nobel Prize in Physiology or Medicine [41]. In one of the more recent patents, Bayer described the hydrogenation of avermectins to ivermectins performed over the RhCl_3_∙H_2_O catalyst in the presence of triphenylphosphine (Scheme 20) [42]. In addition, several successful total syntheses of avermectin and ivermectin by academic research groups have been reported in the literature [43]. However, these syntheses are not economically viable for the production of the drug.

## 10. Rifabutin

**Figure 9:**
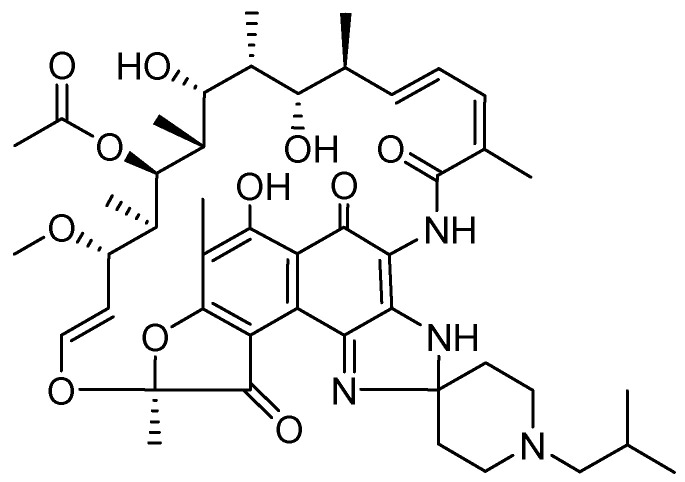
Structural formula of rifabutin.

Rifabutin (Figure 9), a macrocyclic semisynthetic drug of the rifamycin family [44] was developed by the Italian drug company Farmitalia Carlo Erba, and received FDA approval in 1992 for the treatment of tuberculosis, as well as other bacterial infections such as *Mycobacterium avium* complex [45]. The drug works via the inhibition of the DNA-dependent RNA polymerase in bacteria, leading to a suppression of RNA synthesis and cell death [46].

**Scheme 21:**
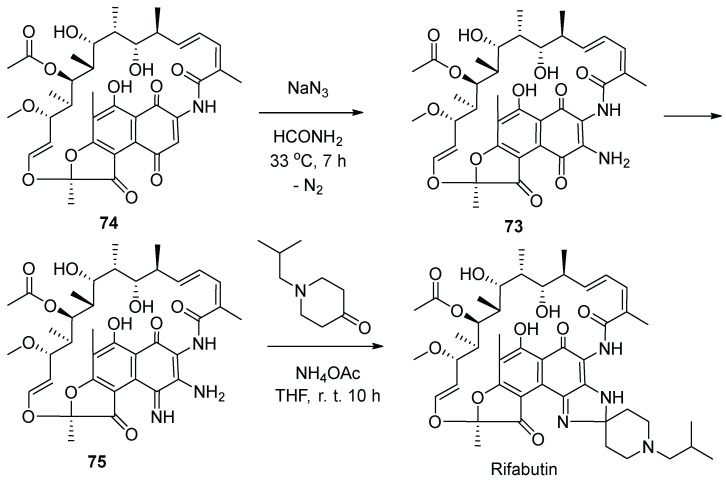
Farmitalia synthesis of rifabutin [30].

The original synthesis of rifabutin was reported by Marsili and colleagues from Framitalia in 1981 [47]. The synthesis relied on 3-aminorifamycin S (**73**) prepared via azidation of rifamycin S (**74**), which was accompanied by a spontaneous loss of nitrogen molecule [48]. Compound **73** was treated with ammonia solution in THF to give 3-amino-4-deoxo-4-iminorifamycin S (**75**). The latter was isolated by crystallization and further involved in cyclocondensation with *N*-isobutyl piperidone, performed at ambient temperature in the presence of ammonium acetate as a mild acidic catalyst. As a result, a spirocyclic imidazoline moiety was installed, and pure rifabutin was isolated without chromatographic purification (Scheme 21).

## 11. Spirapril

**Figure 10:**
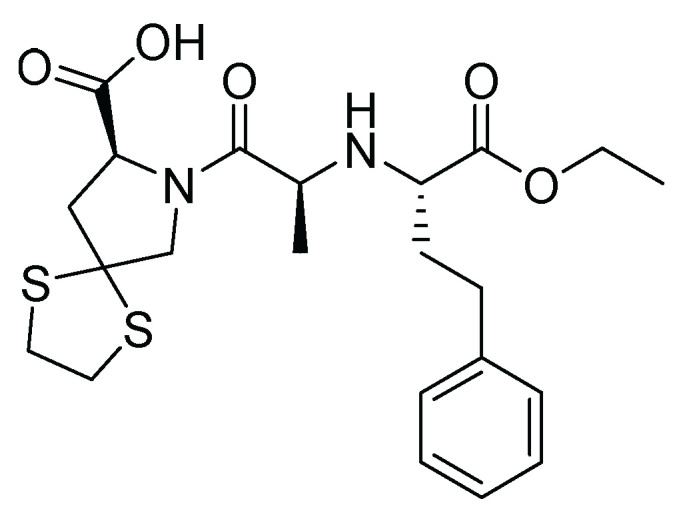
Structural formula of spirapril.

Spirapril (Figure 10), a spirocyclic inhibitor of the angiotensin-converting enzyme (ACE) had been originated and developed by the Schering-Plough Corporation (now Merck & Co.) and was approved in 1995 for the treatment of hypertension. ACE is a part of the renin–angiotensin system regulating blood pressure. Inhibition of ACE suppresses the production of the potent vasoconstrictor angiotensin II from its inactive precursor pro-angiotensin and thus prevents the increase in blood pressure [49]. Spirapril is an ethyl ester prodrug. It undergoes biotransformation to the respective carboxylic acid, spiraprilat, which is the active ACE inhibitor [50].

**Scheme 22:**
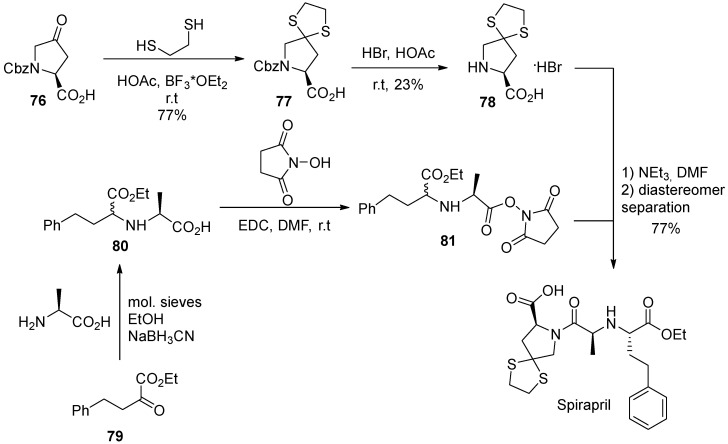
Synthesis of spirapril by the Schering-Plough Corporation [51].

Several syntheses of spirapril have been reported in the patent and periodic literature around the time of the drug’s approval. The original Schering-Plough route was patented in 1984 [51] and later disclosed in greater detail and with a few reaction sequence variants in a 1989 research article [52]. The synthesis commenced with Cbz-protected 4-oxoproline methyl ester (**76**), the carbonyl group of which was protected as spirocyclic thioketal, to give methyl ester **77**. The Cbz group was removed by 20% hydrobromic acid in glacial acetic acid, to give amino acid **78**. The ester side chain building block was prepared by reductive amination of the keto ester **79**. The resulting amino acid **80** was obtained as a mixture of diastereomers. Without separation, the mixture was activated as *N*-hydroxysuccinimide ester (**81**) and reacted with **78** in the presence of triethylamine, to give a good yield of spirapril after diastereomer separation (Scheme 22).

**Scheme 23:**
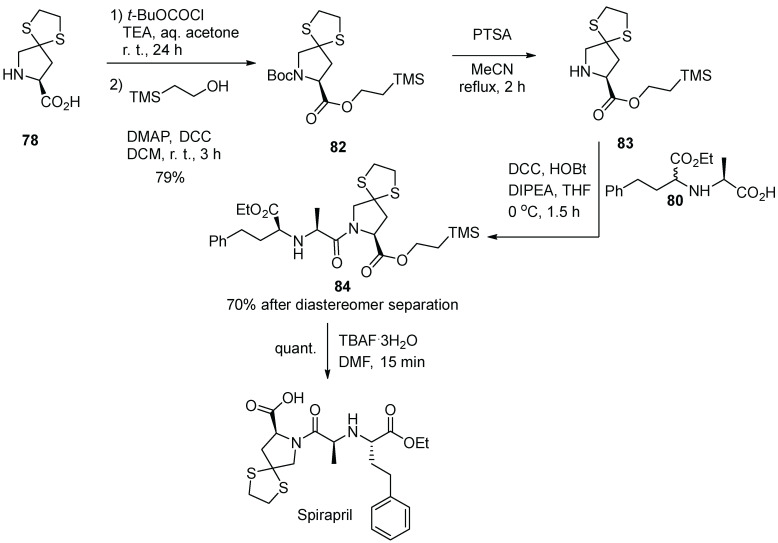
Synthesis of spirapril by the Squibb Corporation [53].

An alternative synthesis reported by the Squibb Corporation (now Bristol Myers Squibb) in 1988 also made use of spirocyclic amino acid **78**. The latter was Boc-protected and the carboxylic function was esterified with 2-(trimethylsilyl)ethanol in the presence of DCC and DMAP to give good yield of ester **82**. The Boc group was then removed by *p*-toluenesulfonic acid (PTSA) and the liberated free pyrrolidine **83** was acylated with carboxylic acid **80** activated by DCC/HOBt. This two-step sequence gave ester **84** in 70% after diastereomer separation. The removal of the 2-(trimethylsilyl)ethyl group with tetrabutylammonium fluoride trihydrate gave spiropril in quantitative yield (Scheme 23) [53].

## 12. Irbesartan

**Figure 11:**
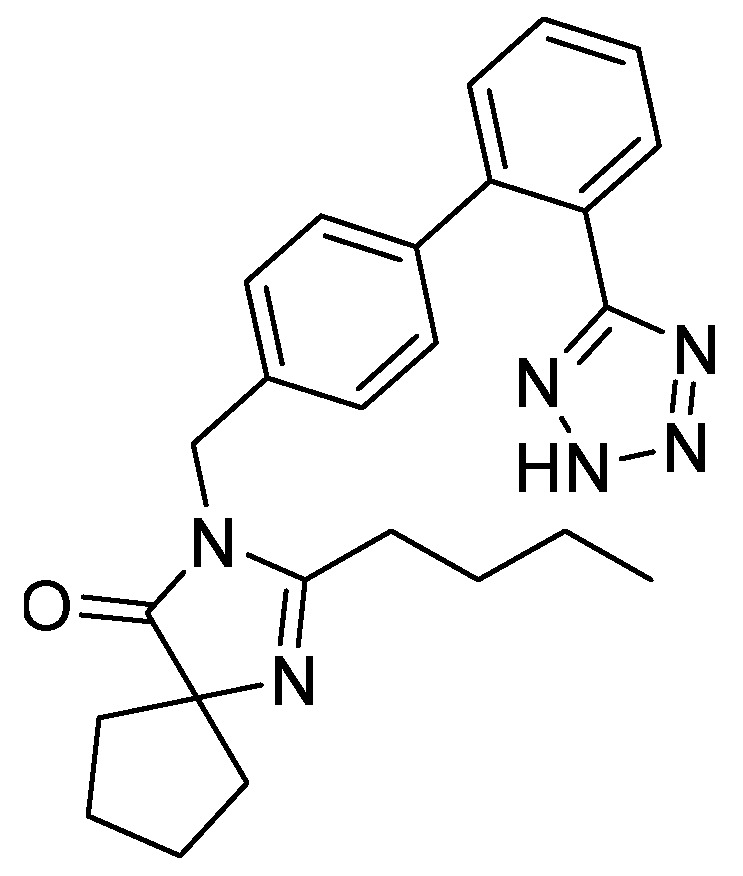
Structural formula of irbesartan.

Irbesartan (Figure 11), a spirocyclic antihypertensive drug was developed by Sanofi Research (now Sanofi-Aventis) and approved for medical use in 1997. The compound is a typical representative of the sartan family of hypertensives, which act by blocking the angiotensin type 1 (AT_1_) receptor and preventing its activation and downstream events leading the blood pressure increase [54]. Irbesartan possesses four key elements of structure which are typical of sartans and could be traced back to losartan, the first-in-class AT_1_ receptor blocker: biphenyl substituted with tetrazole in the *ortho* position of the distal ring, five-membered nitrogen heterocycle (in this case, imidazoline-5-one) and an *n*-butyl side chain [55].

**Scheme 24:**
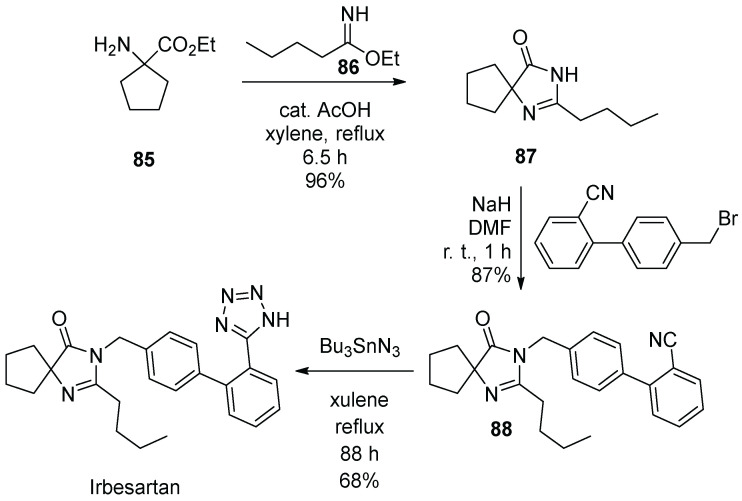
Original route to irbesartan.

The original synthetic route to irbesartan was described in detail in 1995 publication by Bernhart and co-workers (Sanofi Research) [56]. The synthesis relies on commercially available tetramethylene glycine ethyl ester (**85**) as the starting material. Amino ester **85** was condensed with imidate **86** in the presence of a catalytic amount of acetic acid in refluxing xylene to give imidazolin-5-one **87**. The latter was alkylated with 4′-(bromomethyl)-[1,1′-biphenyl]-2-carbonitrile and the resulting derivative **88** was treated with tributyl tin azide in refluxing xylene, to give irbesartan (Scheme 24).

**Scheme 25:**
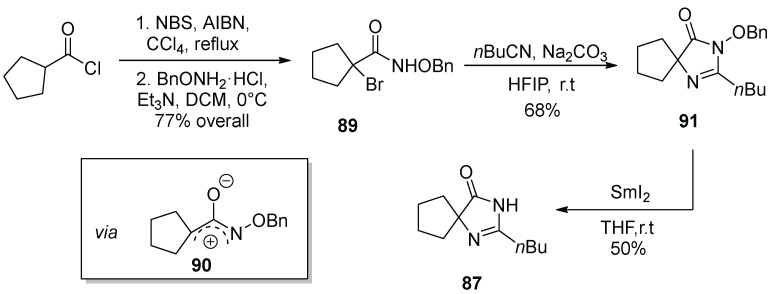
Synthesis of key building block **87**.

Interestingly, the key spirocyclic imidazolin-5-one building block **87** was recently accessed by Wu and DiPoto via a novel annulation of azaoxyallyl cations. Cyclopentanoyl chloride was first transformed into *O*-benzyl hydroxamic acid **89**. The latter was treated with the base to generate azaoxyallyl cation **90**, which reacted with pentanenitrile to give benzyloxy-substituted imidazolin-5-one **91**. *N*-Deoxygenation with samarium iodide gave **87**, thereby completing the formal synthesis of irbesartan (Scheme 25) [57].

**Scheme 26:**
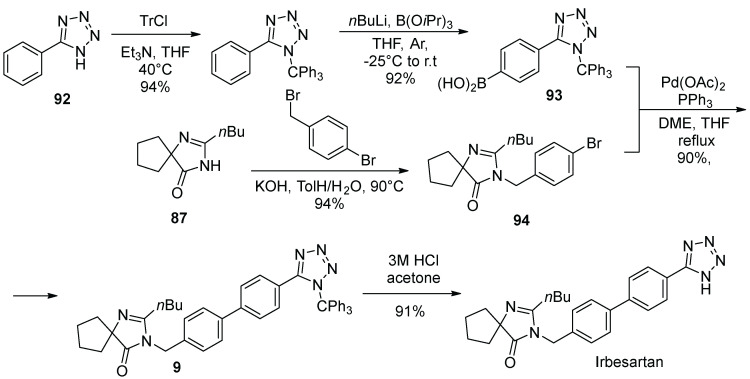
Synthesis of irbesartan by Israeli group.

Intermediate **87** was also key in the synthesis of irbesartan patented by a group of Israeli scientists in 2004 [58]. The convergent route started with *C*-phenyl tetrazole (**92**), which was protected with a trityl group and borylated in the *para* position via lithiation to give boronic acid **93**. In parallel, intermediate **87** was *N*-alkylated with *p*-bromobenzyl bromide and the resulting intermediate **94** was involved in Suzuki coupling with boronic acid **93**, to give trityl-protected irbesartan **95**. Removal of the trityl group furnished the target molecule (Scheme 26).

**Scheme 27:**
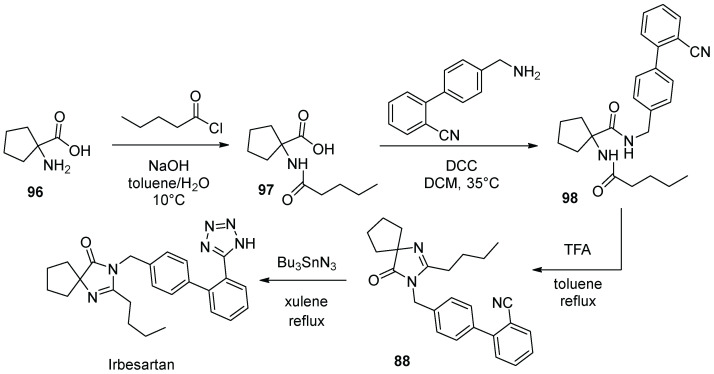
Dr. Reddy’s Laboratories’ synthesis of irbesartan [59].

Among the synthetic routes to irbesartan featured in the patent literature, the following synthesis from Dr. Reddy’s Laboratories is notable. Similarly to the Sanofi route (vide supra), the synthesis started with tetramethylene glycine (**96**), which was acylated with pentanoyl chloride under phase transfer conditions to give carboxylic acid **97** which, in turn, was amidated with 4′-(aminomethyl)-[1,1′-biphenyl]-2-carbonitrile to give the ‘fully decorated’ precursor **98**. The latter was cyclodehydrated in refluxing toluene in the presence of trifluoroacetic acid, to give nitrile **88** and, after [3 + 2] cycloaddition with tributyltin azide, irbesartan (Scheme 27) [59].

**Scheme 28:**
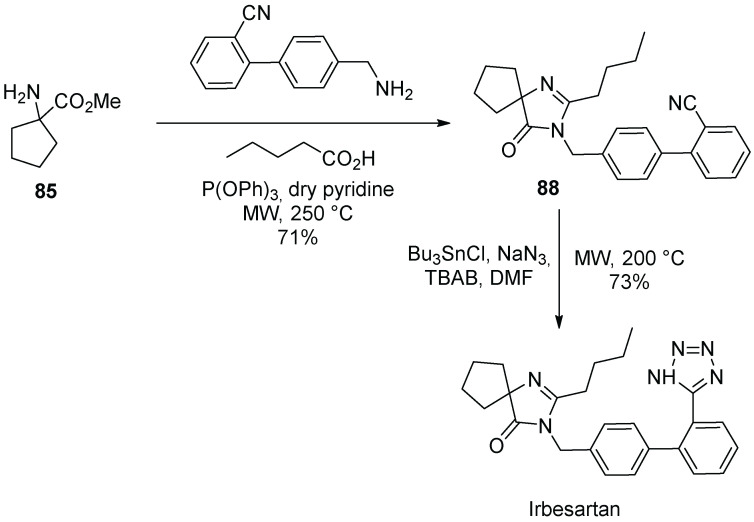
ArQule synthesis of irbesartan [60].

An approach rather similar in spirit to the Dr. Reddy’s route was described in 2006 by ArQule scientists [60]. It is based on the three-component, microwave-promoted cyclodehydrative synthesis of irbesartan’s key precursor **88** from ester **85**, 4′-(aminomethyl)-[1,1′-biphenyl]-2-carbonitrile and pentanoic acid, in the presence of triphenoxyphosphine. The conversion of nitrile **88** to irbesartan was achieved via hte in situ generation of tributyltin azide (Scheme 28).

**Scheme 29:**
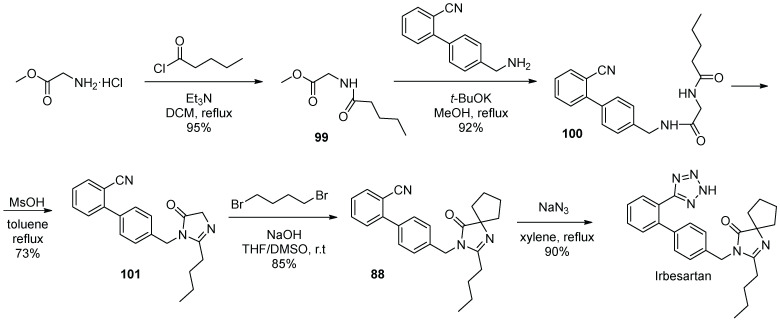
Beijing University of Chemical Technology route to irbesartan.

Finally, a fundamentally different approach to irbesartan was developed in 2022 at Beijing University of Chemical Technology [61]. In this route, the spirocyclic scaffold of the drug was elaborated at a more advanced stage of the synthesis, as shown in Scheme 29. Glycine methyl ester was acylated by pentanoyl chloride and ester **99** was amidated with 4′-(aminomethyl)-[1,1′-biphenyl]-2-carbonitrile under forcing, base-promoted conditions, to give diamide **100**. The latter was cyclodehydrated in refluxing toluene in the presence of methanesulfonic acid to give intermediate **101**, which was found to be sufficiently C–H acidic to be doubly alkylated with 1,4-dibromobutane to furnish spirocyclic imidazoline-5-one **88**. Notably, the nitrile-to-tetrazole conversion was achieved without the use of the toxic tin reagent (used in all previously described syntheses), which makes this 2022 route quite environmentally friendly and amenable to the plant-scale production of the drug.

## 13. Cevimeline

**Figure 12:**
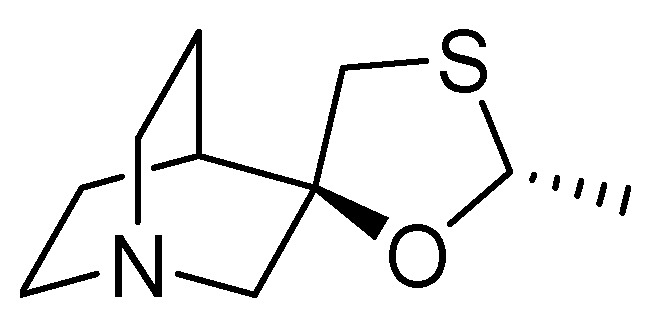
Structural formula of cevimeline.

Cevimelin (Figure 12), a spirocyclic derivative of quinuclidine acts as an agonist of muscarinic M_1_ and M_3_ receptors [62]. Originating from the Israel Institute of Biological Research, the drug was developed by the Daiichi Sankyo company and gained regulatory approval in 2000 for the treatment of dry mouth and Sjögren’s syndrome [63]. Recently, the drug has been investigated in such therapeutic areas as cognitive impairment and neurodegenerative diseases [64].

**Scheme 30:**
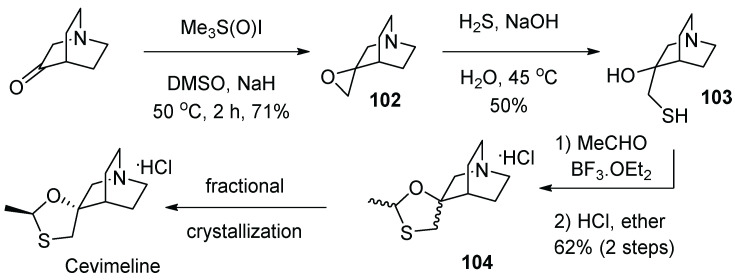
Original synthesis of cevimeline.

In the original synthesis of cevimeline patented by the Israeli group [65], commercially available quinuclidin-3-one was converted to epoxide **102** using the Corey–Chaykovsky reaction with trimethyloxosulfonium iodide (Me_3_S^+^(O)I^−^) and sodium hydride. The resulting epoxide **102** was treated with hydrogen sulfide in a basic aqueous medium, which led to epoxide opening and the formation of hydroxy thiol **103**. The latter was subjected to thio/oxa acetal formation on treatment with acetaldehyde in the presence of boron trifluoride etherate. Cevimeline contained in the diastereomeric mixture of products **104** was separated by fractional crystallization (Scheme 30). The patent [65] presented a rather elaborate scheme for the fractional crystallization.

**Scheme 31:**
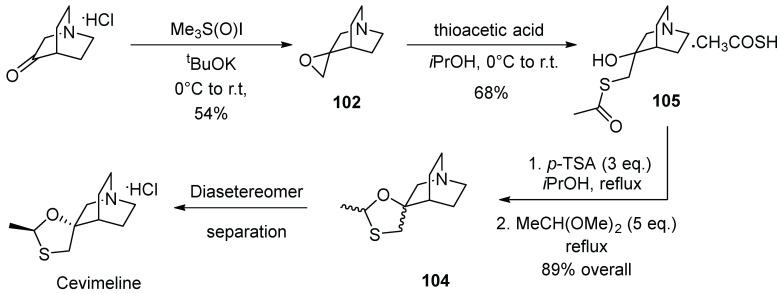
Cevimeline synthesis by Apotex Pharmachem, Inc. [66].

In 2008, the Canadian company Apotex Pharmachem, Inc. filed a patent application for an improved synthesis of cevimeline which is similar in spirit to the original Israeli route. The Corey–Chaykovsky epoxidation of quinuclidine-3-one was performed with potassium *tert*-butoxide, and the opening of epoxide **102** was achieved with thioacetic acid in lieu of hydrogen sulfide gas. Thioacetate **105** was condensed directly with acetaldehyde dimethyl acetal, which gave an excellent yield of the diastereomeric mixture **104**, from which cevimeline was isolated by diastereomer separation (Scheme 31) [66].

**Scheme 32:**
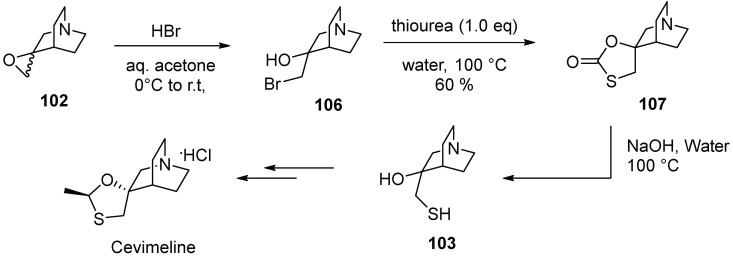
Cevimeline synthesis by Emcure Pharmaceuticals, Ltd. [67].

In 2013, the Active Pharmaceutical Ingredient R&D Center of the Indian company Emcure Pharmaceuticals, Ltd. published an even further improved route to cevimeline, in which safe and odorless thiourea is utilized as a thiolating reagent. Epoxide **102** was first converted to hydroxy bromide **106**, which was then refluxed with thiourea in an aqueous medium to furnish thiolactone **107**. The latter was hydrolyzed with aqueous sodium hydroxide to give the key hydroxy thiol **103**, which was converted to cevimeline using the same routine as was developed in the original synthesis (vide supra) (Scheme 32) [67].

**Scheme 33:**
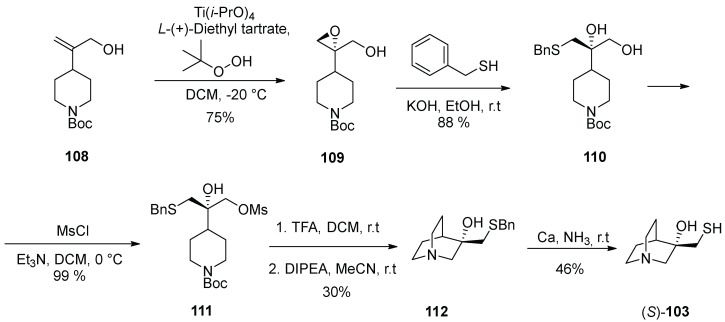
Synthesis of key intermediate (*S*)-**103** by F. Hoffmann-La Roche, Ltd. [68].

An intriguing, stand-alone approach to enantiomerically pure hydroxy thiol (*S*)-**103** was published by F. Hoffmann-La Roche, Ltd. six years prior to the approval of cevimeline for medical use [68]. In this synthesis, the quinuclidine nucleus was elaborated quite late into the synthetic route. The synthesis relied on olefin **108** synthesized from pyridine-4-carboxaldehyde in a few trivial steps. Sharpless epoxidation gave enantiomerically pure (ee 94%) epoxide **109**, which was opened with benzyl mercaptan to give diol **110**. Mesylation of the primary hydroxy group set the scene for the intramolecular S*_N_*2 reaction to form the quinuclidine core. This was achieved by the removal of the Boc group from the piperidine nitrogen atom in **111** and by triggering the nucleophilic substitution by DIPEA. The bnzyl group was removed from intermediate **112** by dissolving the metal (Ca) reduction in ammonia (Scheme 33).

## 14. Eplerenone

**Figure 13:**
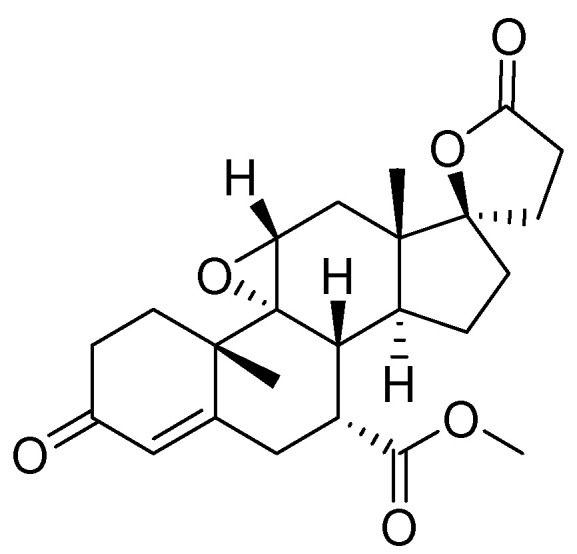
Structural formula of eplerenone.

Eplerenone (Figure 13), a spirocyclic antagonist of mineralocorticoid receptors developed by Ciba-Geigy was approved in 2002 for the treatment of hypertension and heart failure [69]. It is related to spironolactone but displays better selectivity to its primary target through the affinity to androgen, progestogen, glucocorticoid, or estrogen receptors and thus is devoid of undesired sexual side effects typical of spironolactone, which limit its use [70].

**Scheme 34:**
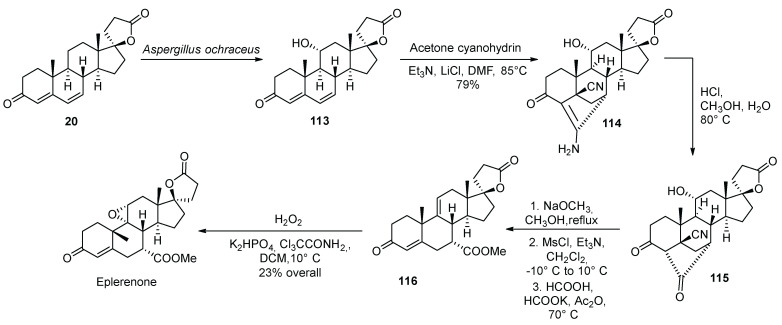
Synthesis of eplerenone by Searle & Co. [71].

Among industrial syntheses of eplerenone, the route patented in 1998 by Searle & Co. is quite notable [71]. The synthesis starts with steroid intermediate **20** (also useful in the synthesis of spironolactone, vide supra) which is subjected to regiospecific and stereoselective hydroxylation by fermentation with the *Aspergillus ochraceus* mold. The hydroxy group in **113** serves as a kind of masked double bond which is unmasked later in the synthesis. Condensation with two equivalents of cyanide (in the form of acetone cyanohydrin) results in the formation of polycyclic enamine **114**. The latter is hydrolyzed to ketone **115**, which is then put through a sequence of steps including treatment with sodium methoxide (opening the ketone linkage as the methyl ester with simultaneous elimination of the cyanide), mesylation of the hydroxy group, and elimination of methane sulfonic acid to give the olefinic intermediate **116**. The latter is epoxidized on treatment with hydrogen peroxide in a phosphate buffered solution, to give eplerenone (Scheme 34).

**Scheme 35:**
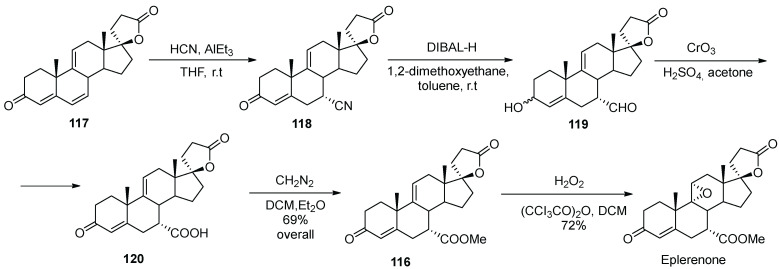
Synthesis of eplerenone by Ciba-Geigy [72].

In 1997, Ciba-Geigy chemists published their synthetic route to eplerenone, which basically provides another approach to the synthesis of intermediate **116** starting from the spirocyclic steroid lactone **117**. Conjugate addition of hydrogen cyanide at the δ-position proceeded stereoselectively and gave the intermediate **118**. Reduction with DIBAL-H gave aldehyde alcohol **119**, which was oxidized into keto carboxylic acid **120**. Esterification with diazomethane gave the intermediate **116** (at which point the overall yield over four steps was a remarkable 69%). Finally, epoxidation with hydrogen peroxide gave eplerenone (Scheme 35) [72].

**Scheme 36:**
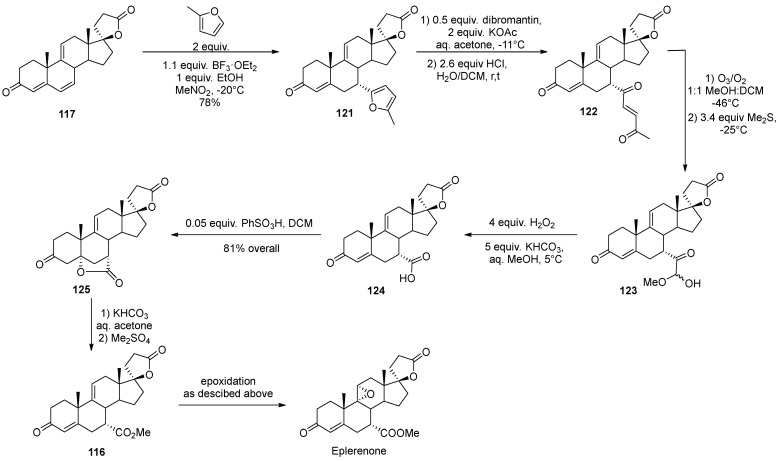
Synthesis of eplerenone by Pfizer [73].

An interesting eplerenone synthesis was published in 2006 by Pfizer’s process chemistry team [73]. The synthesis commences with the same spirocyclic lactone **117** as was employed by Ciba-Geigy (vide supra). 2-Methylfuran was employed as a chemical equivalent of the carboxylic group. On Lewis acid catalysis, it underwent arylation of the distal conjugated double bond. The furan moiety in **121** was hydrolyzed to enedione **122** and the latter, without isolation, was ozonolyzed to give the hemiacetal **123**. The latter was subjected to Beyer–Villiger oxidation, to give carboxylic acid **124**, which was forced to close to isolable bis-lactone **125**. The strained lactone in **125** was opened up via elimination and the intermediate carboxylic acid was esterified with methyl sulfate to give ester **116**. The latter is only one epoxidation step away from eplerenone (Scheme 36).

**Scheme 37:**
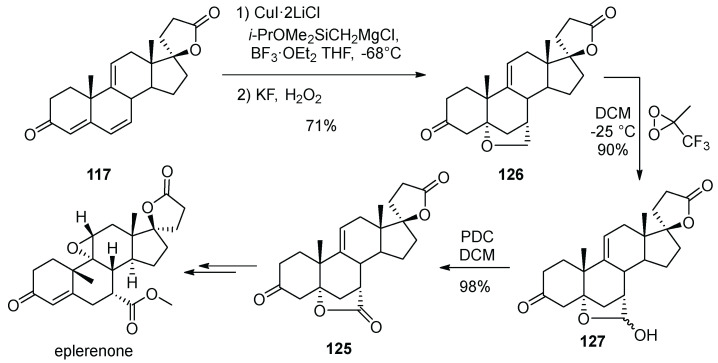
Synthesis of eplerenone by Chinese Academy of Sciences [74].

Intermediate **125** is clearly key to the large-scale synthesis of eplerenone. In 2011, a Chinese Academy of Sciences group published an interesting approach to it from triene **117**, which involves the conjugate addition of silyl methyl cuprate, Tamao oxidation and another intramolecular oxa-Michael addition, to give cyclic ether **126**. Curiously, methyltrifluoromethyl dioxirane was found to selectively oxidize ether **126** to lactol **127**. PDC oxidation of the latter gave a nearly quantitative yield of lactone **125**, which could now be opened and epoxidized to eplerenone as described by Pfizer (vide supra) (Scheme 37) [74].

## 15. Drospirenone

**Figure 14:**
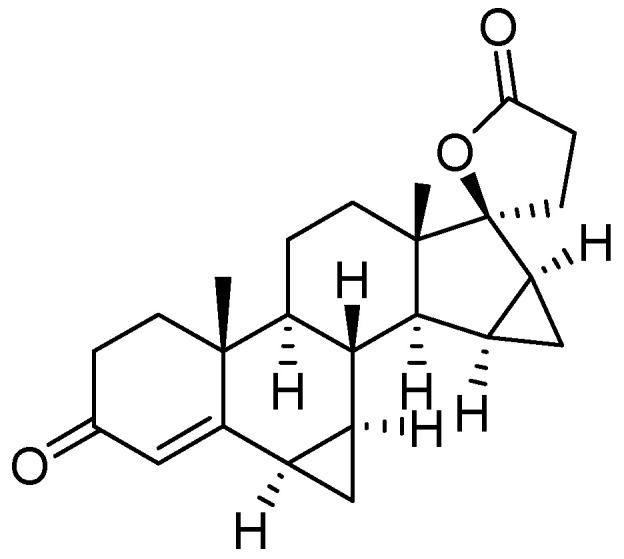
Structural formula of drospirenone.

Drospirenone (Figure 14) is another steroid spirocyclic lactone drug structurally related to spironolactone and eplerenone, antagonists of mineralocorticoid receptors. However, pharmacologically, drospirenone is different from the latter two drugs. In addition to having a high affinity to mineralocorticoid receptors, it binds rather tightly to progesterone receptors and less tightly to androgen receptors [75], which underlies its use as a birth control medication approved in 2000 [76].

**Scheme 38:**
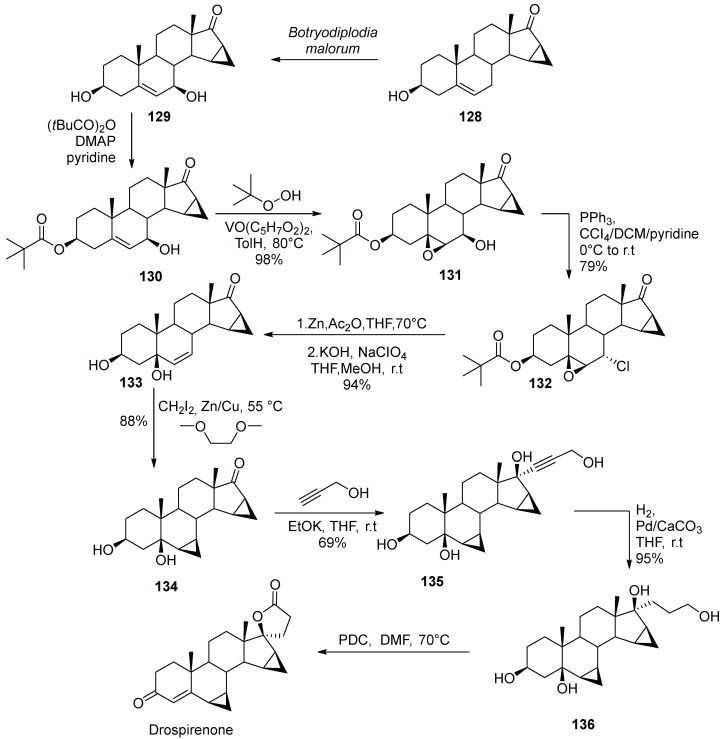
Synthesis of drospirenone by Schering AG [77].

The earliest synthesis of drospirenone was published by Schering AG in 1982 [77]. The synthesis commenced with compound **128**, obtained from 3β-hydroxy-5,15-androstadien-17-one by Corey cyclopropanation. Microbial hydroxylation of **128** gave diol **129**, which was mono-pivaloylated at the less-hindered hydroxyl group, to give allylic alcohol **130**. The olefinic portion of **130** was epoxidized with *tert*-butyl hydroperoxide under vanadium(IV) oxide-acetylacetonate catalysis. The resulting epoxide **131** was converted to chloride **132** by treatment with Ph_3_P/CCl_4_ in pyridine. Reductive elimination of the chlorine atom on treatment with zinc in acetic acid, followed by saponification, produced the diol **133**. The latter was subjected to Simmons–Smith cyclopropanation, to establish the basic core of the target compound (**134**). Now the scene was set to establish the spirocyclic lactone moiety. Propargylic alcohol was doubly deprotonated and added to the ketone portion of **134**, to give propargylic tetraol **135**. The triple bond in **135** was hydrogenated over calcium carbonate. The resulting compound **136** was treated with PDC, which triggered three events: oxidation of the primary alcohol moiety to carboxylic acid and lactonization, oxidation of the secondary alcohol to ketone, and dehydration, to form the α,β-unsaturated system of drospirenone (Scheme 38).

**Scheme 39:**
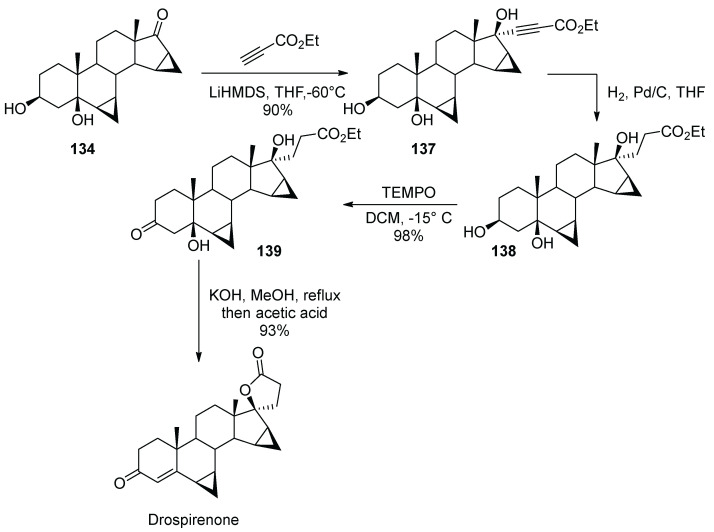
Evestra, Inc. synthesis of drospirenone [78].

An approach similar in spirit to the synthesis of drospirenone by Schering AG was patented in 2010 by Evestra, Inc. [78]. The synthesis starts with bis-cyclopropane **134**, to which the *C*-anion of ethyl propiolate was added to the carbonyl group. Hydrogenation of the triple bond in the resulting intermediate **137** gave triol **138**, in which the secondary alcohol moiety was oxidized by TEMPO. Treatment of ketone **139** with methanolic potassium hydroxide followed by acidification with acetic acid led to the formation of spirocyclic lactone and the dehydration of the β-hydroxy cyclohexanone moiety, which resulted in drospirenone (Scheme 39).

**Scheme 40:**
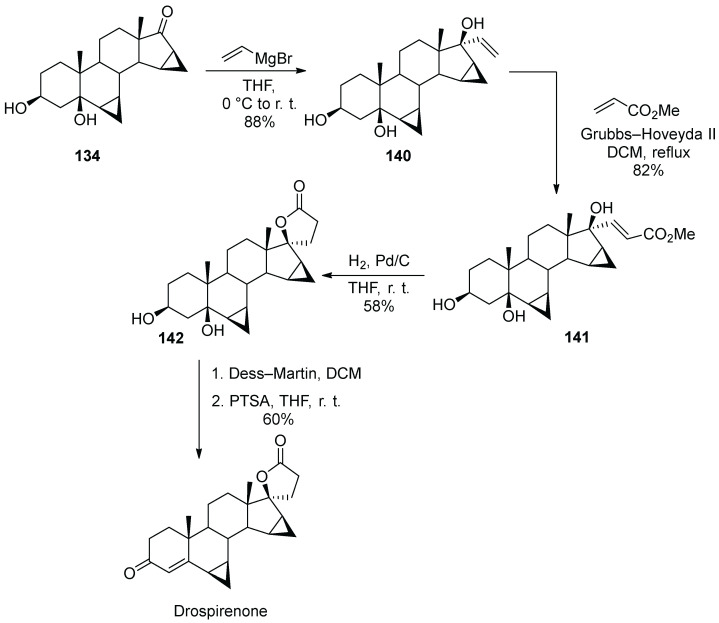
University of Bologna synthesis of drospirenone [79].

A different approach to the construction of drospirenone’s spirocyclic lactone motif was reported in 2008 by a University of Bologna group [79]. Ketone **134** was treated with excess vinyl magnesium bromide. The resulting tertiary allylic alcohol **140** was involved in the olefin metathesis reaction with methyl acrylate catalyzed by the second-generation Hoveyda–Grubbs catalyst. The resulting intermediate **141**, having all the requisite carbon atoms necessary for the formation of spirocyclic γ-lactone, was hydrogenated over palladium on charcoal, which resulted in the reduction of the double bond and the lactone ring closure, to give **142**. Final steps toward drospirenone were the Dess–Martin oxidation of the secondary alcohol moiety and the dehydration of the resulting ketone (Scheme 40).

## 16. Ledipasvir

**Figure 15:**
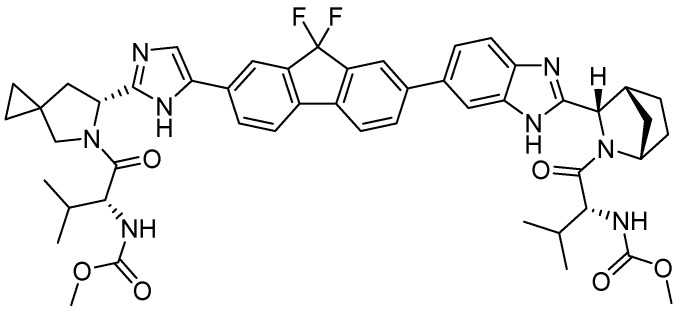
Structural formula of ledipasvir.

Ledipasvir (Figure 15), an inhibitor of the hepatitis C virus non-structural protein 5A (HCV NS5A) was developed by Gilead Sciences and was approved in 2014 for the treatment of genotype 1 HCV in combination with sofosbuvir [80]. The pertinence of this drug to the present review is determined by the spirocyclic pyrrolidine moiety in its structure.

**Scheme 41:**
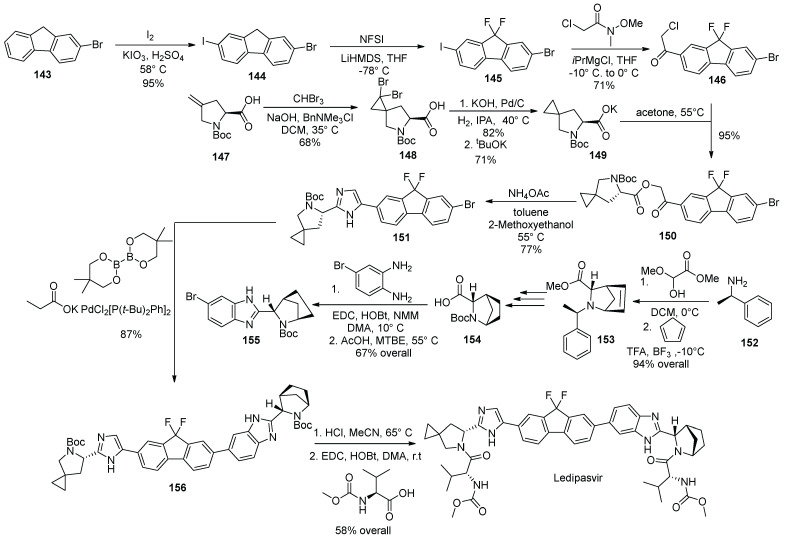
Gilead Sciences’ synthesis of ledipasvir [81].

The complexity of ledipasvir’s structure mandates a lengthy synthetic route to assemble the drug molecule. The original synthesis patented by Gilead in 2013 [81] is presented in Scheme 41. The synthetic scheme includes the assembly of the requisite building blocks from four different directions, thereby being remarkably convergent. From one direction, 2-bromofluorene (**143**) was iodinated in position 7, to give **144**. The methylene group in the latter was bis-fluorinated with *N*-fluorobenzenesulfonimide (NFSI) to give compound **145**, which was converted to an aryl magnesium species at the more reactive, iodo side and coupled with chloroacetic acid Weireb amide to give building block **146**. From another direction, Boc-protected 4-methylene *L*-proline (**147**) was converted to spiro dibromo cyclopropane **148**, which was dibrominated and converted to potassium carboxylate **149**. The latter was involved in a nucleophilic substitution reaction with the chloroacetyl compound **146** to give keto ester **150**, which was reacted with ammonium acetate to give the imidazole **151**. The assembly of the bicyclic pyrrolidine portion of ledipasvir started with enantiopure α-methyl benzylamine (**152**,) which was used to form a Schiff base with methyl glyoxylate hemiacetal. The Schiff base was involved in a Lewis acid-catalyzed Diels–Alder reaction with cyclopentadiene, and the desired bicyclic structure was established (compound **153**). Three trivial transformations (debenzylation with double bond reduction, Boc-protection and ester hydrolysis) gave carboxylic acid **154**, which was employed in benzimidazole synthesis to give compound **155**. Now the two halves of the ledipasvir molecule (**151** and **155**) were brought together via a Suzuki coupling involving the in situ formation of the aryl boronic acid intermediate. The resulting intermediate **156** was deprotected and acylated at both liberated nitrogen atoms with *N*-methoxycarbonyl valine, to give lidepasvir.

**Scheme 42:**
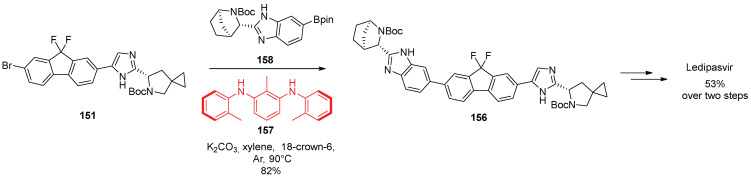
Palladium-free Suzuki–Miyaura-type coupling towards ledipasvir.

The Gilead Sciences route to ledipasvir may well be quite optimal, considering that there have not been any attempts to improve it as judged by the literature on the subject. However, in 2021, a Suzuki–Miyaura coupling of the two halves of the ledipasvir molecule was selected as a drug synthesis example to illustrate the power of the novel aryl halide to aryl boronic acid coupling protocol where no palladium catalyst is employed. The two research groups from China reported the use of the organocatalyst **157** to bring about the coupling of the aryl bromide **151** with the boronic acid pinacol ester **158**. Considering the high yield of the advanced ledipasvir precursor **156** reported for this coupling, this palladium-free approach appears much more amenable to large-scale pharmaceutical production than the original Gilead Sciences route (Scheme 42) [82].

## 17. Rolapitant

**Figure 16:**
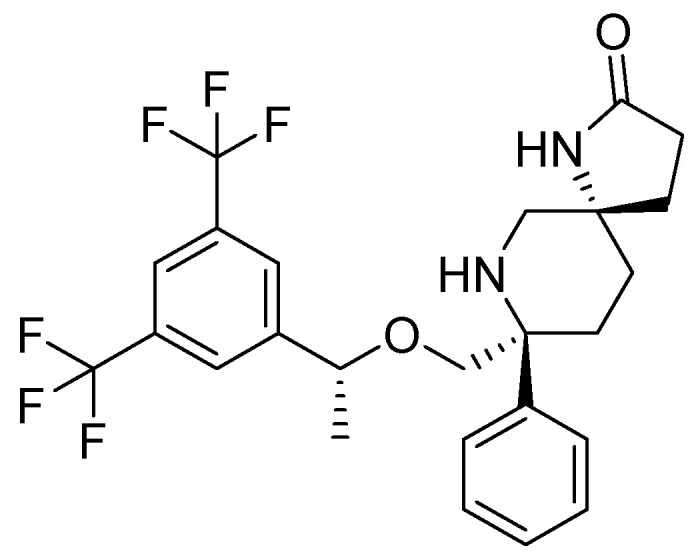
Structural formula of rolapitant.

Rolapitant (Figure 16), a spirocyclic neurokinin NK1 receptor antagonist comprising three stereogenic centers originated in the laboratories of Schering-Plough, and was approved for the treatment of chemotherapy-induced nausea and vomiting in 2015 [83]. The drug’s syntheses are primarily featured in the patent literature.

**Scheme 43:**
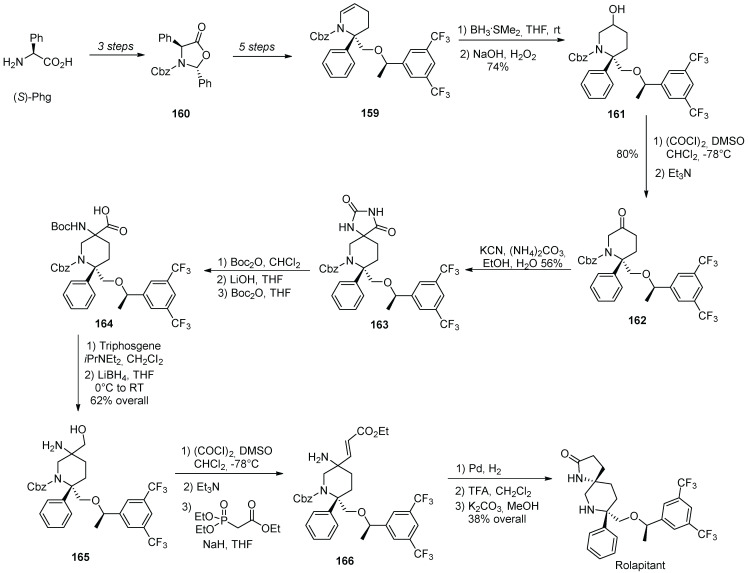
Schering-Plough synthesis of rolapitant [84].

The original synthesis of rolapitant patented by Schering-Plough [84] relies on the supply of the key building block **159**. Synthetically, the latter can be traced back to (*S*)-phenylglycine over eight synthetic operations, including the preparation of oxazolidinone **160**, as detailed further below. Cyclic enamine **159** was subjected to standard hydroboration–oxidation conditions, to afford alcohol **161**, which was oxidized to ketone **162** under the Swern conditions. Ketone **162** was involved in a standard hydantoin synthesis with potassium cyanide and ammonium carbonate, to afford spirocyclic compound **163**. The latter was Boc-protected, hydrolyzed and Boc-protected again to give α-amino acid **164**. The carboxylic group in **164** was reduced via a mixed anhydride to give amino alcohol **165** which was oxidized (again under the Swern conditions) and involved, without protection of the amino group, in a Horner–Wadsworth–Emmons olefination, to give the acrylate ester **166**. The latter turned out to be a direct precursor of rolapitant, and was converted to the drug by hydrogenation (accompanied by lactam formation) and protecting group removal (Scheme 43).

**Scheme 44:**
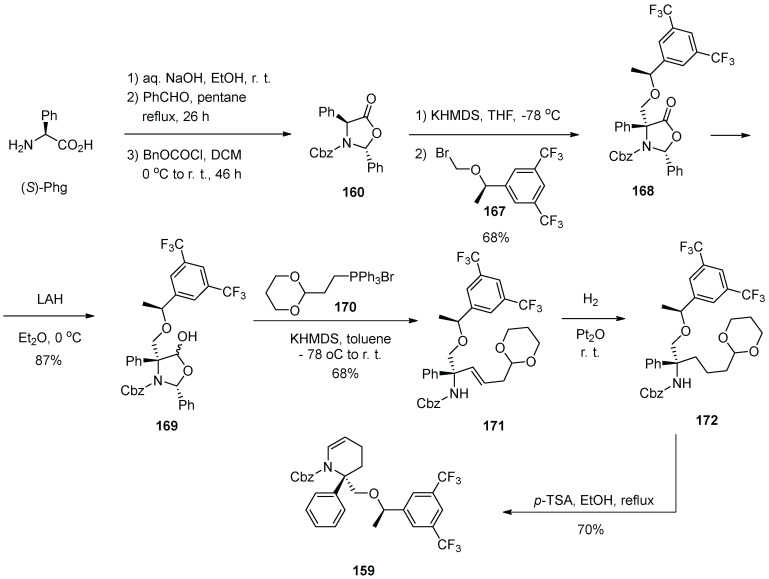
Preparation of key building block **159** for the synthesis of rolapitant.

As to the synthesis of key building block **159** from (*S*)-phenylglycine ((*S*)-Phg), it was accomplished as follows. (*S*)-Phg was converted to diastereomerically pure oxazolidinone **160** on condensation with benzaldehyde and Cbz-protection [85]. Oxazolidinone **160** was *C*-alkylated in diastereoselective fashion with chiral bromide **167**. The carbonyl group of the resulting intermediate **168** was reduced by lithium alumohydride, and the lactol **169** was obtained. The aldehyde form of the latter reacted with phosphonium ylide generated from the alkyl phosphonium bromide **170**, and the elaborate olefinic building block **171** was obtained. It was hydrogenated over platinum dioxide and the resulting acetal **172** was treated with *p*-toluene sulfonic acid in refluxing ethanol. This led to the removal of the acetal protecting group and the cyclization of the Cbz-protected amino group onto the liberated aldehyde carbonyl group, to give the target key intermediate **159** (Scheme 44) [84].

**Scheme 45:**
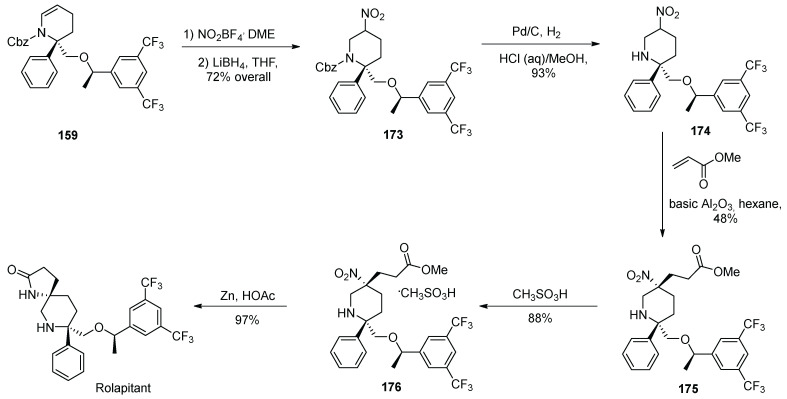
A 2013 Opko Health, Inc. synthesis of rolapitant [86].

In 2013, Opko Health, Inc. patented an alternative route to rolapitant which is also based on the key building block **159** [86]. This intermediate was nitrated and reduced by lithium borohydride to give the nitro alkane **173**. Removal of the Cbz group (to give **174**) and subsequent alkylation of the nitro alkane portion with methyl acrylate over basic alumina gave compound **175**, which was converted to the methanesulfonic acid salt **176**, for easy isolation. The reduction of **176** with zinc metal in acetic acid triggered lactamization and gave rolapitant (Scheme 45). Thus, the Opko Health, Inc. synthesis appears to be shorter than the original Schering-Plough route and more amenable to large scale API production.

**Scheme 46:**
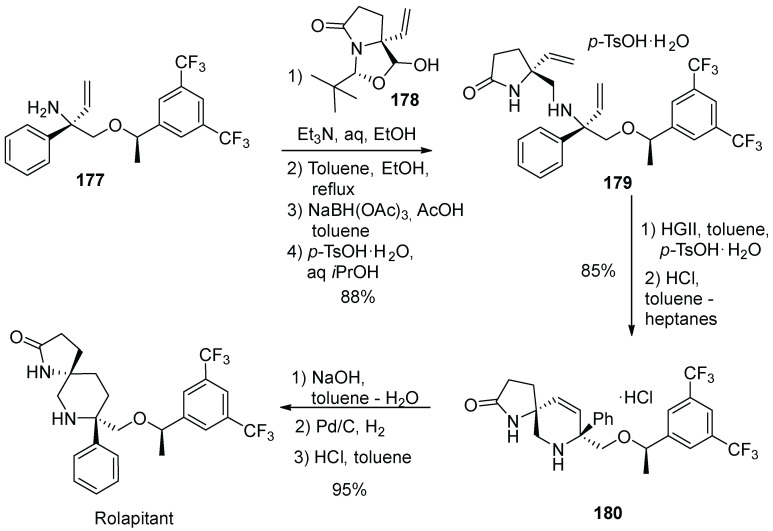
A 2016 Opko Health, Inc. synthesis of rolapitant [87].

In 2016, Opko Health, Inc. patented a fundamentally different route to rolapitant which relied on the chiral building block **177** (for the sake of brevity, we do not discuss its synthesis) [87]. It was reductively alkylated with the masked aldehyde building block **178** to give the bis-olefinic compound **179**. In the latter, a scene was set for intramolecular olefin metathesis, which was successfully brought about using the Hoveyda–Grubbs second-generation ruthenium catalysts (HGII), and the resulting spirocycle **180** was isolated as hydrochloride salt. The latter advanced intermediate was free-based by aqueous sodium hydroxide and hydrogenated over palladium on charcoal, to give rolapitant (Scheme 46).

## 18. Apalutamide

**Figure 17:**
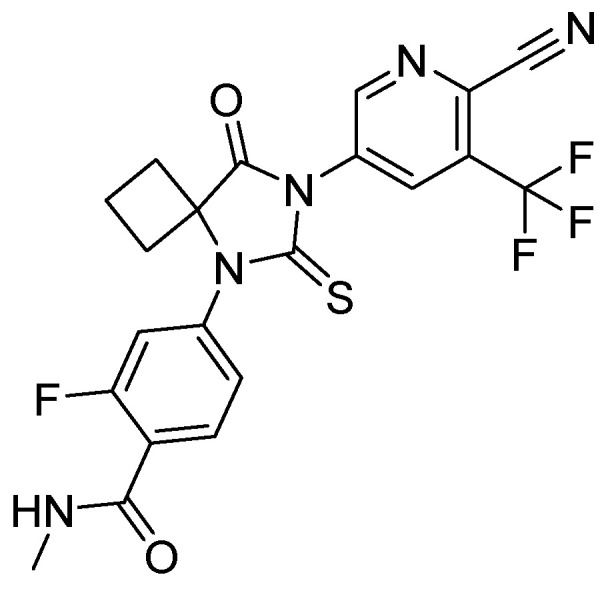
Structural formula of apalutamide.

**Scheme 47:**
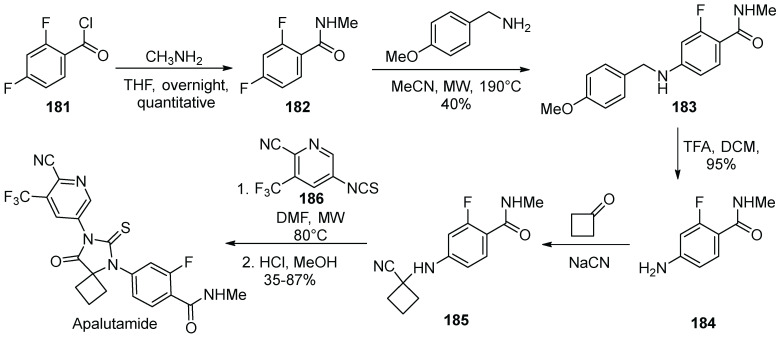
University of California original synthesis of apalutamide.

Apalutamide (Figure 17), a spirocyclic androgen receptor antagonist discovered by the Sawyers/Jung team at UCLA was approved for the treatment of prostate cancer in the US in 2018 and in Europe in 2019 [88]. The original synthesis was patented in 2007 by the discovery team [89]. It started with the benzoyl chloride **181**, which was reacted with methylamine and the resulting amide **182** was involved in an aromatic nucleophilic substitution reaction with *p*-methoxybenzylamine, to give **183**. Upon removal of the PMB group, aniline **184** was involved in a Strecker reaction with cyclobutanone, to give nitrile **185**. The monothiohydantoin ring of apalutamide was formed on microwave-promoted condensation with the thioisocyanate **186**, followed by acidic hydrolysis on the initially formed imine (Scheme 47).

**Scheme 48:**
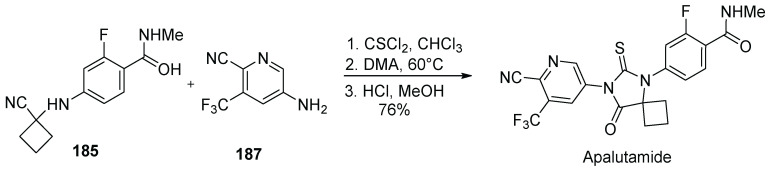
Apalutamide synthesis with thiophosgene from Sloan Kettering [90].

Later, a Sloan Kettering team patented an apalutamide synthesis in which thiohydantoin formation was performed via the in situ generation of the thioisocyanate **186** from the aniline **187** by reacting the latter with Strecker nitrile **185** in the presence of thiophosgene (Scheme 48) [90].

**Scheme 49:**
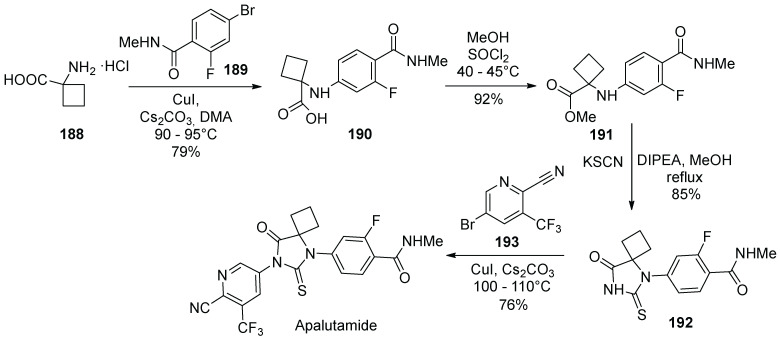
Apalutamide synthesis by Hangzhou Cheminspire Technologies Co., Ltd [91].

A number of different approaches to apalutamide, mostly focused on the synthesis of pyridine-3-amine **187** were disclosed [88]. A somewhat different approach to apalutamide assembly which involves two copper(I)-catalysed *N*-arylation steps was patented in 2018 by Hangzhou Cheminspire Technologies Co., Ltd. (Hangzhou, China) [91]. The synthesis avoided the use of thiophosgene or thiophosgene equivalents by generating the thiohydantoin using potassium thiocyanate. Cyclobutane α-amino acid **188** was used as a partner in the copper(I)-catalyzed reaction with aryl bromide **189**, to give carboxylic acid **190**. Following esterification, compound **191** was reacted with potassium thiocyanate to give the thiohydantoin portion of apalutamide (**192**). from which the target drug molecule was crafted via copper(I)-catalyzed arylation with 3-bromopyridine **193** (Scheme 49).

## 19. Moxidectin

**Figure 18:**
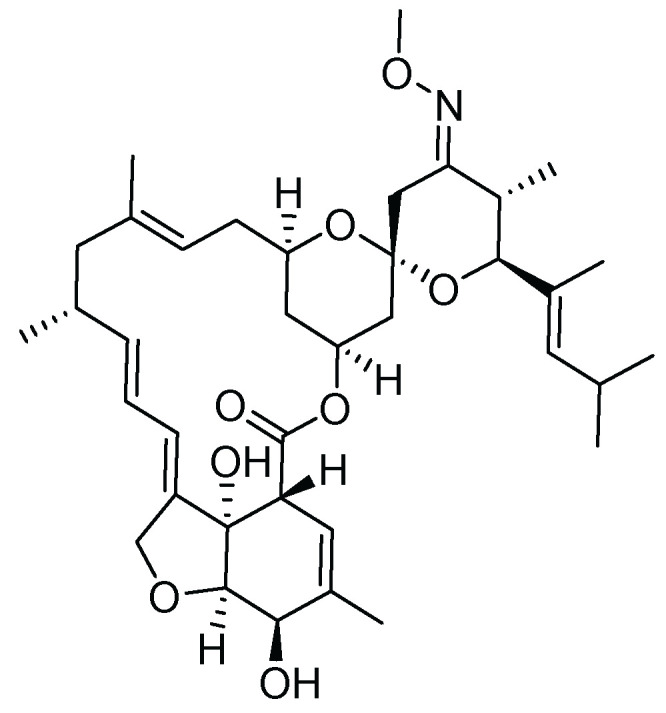
Structural formula of moxidectin.

Moxidectin (Figure 18), a macrolide antiparasitic drug containing an (*S*)-1,7-dioxaspiro[5.5]undecane motif was derived from *Streptomyces* bacteria discovered in the late 1980s in an Australian soil sample [92]. It was initially employed for the treatment of helminths in house animals. However, in 2018, moxidectin received FDA approval to treat onchocerciasis (river blindness) in patients aged 12 and older [93]. Moxidectin exerts its antiparasitic action by selectively binding to the parasite’s GABA-A and glutamate-gated chloride ion channels [94].

**Scheme 50:**
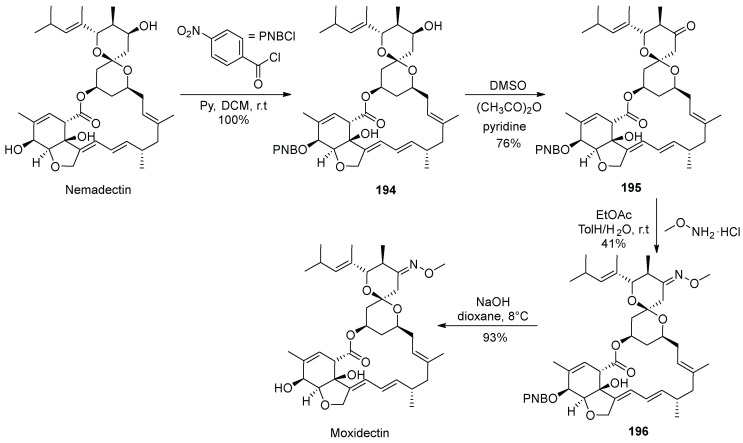
Semisynthesis of moxidectin from nemadectin.

Chemically, moxidectin belongs to the milbemycin class of macrocyclic lactones. It is obtained by the semisynthetic modification of nemadectin (F-29249α) which, in turn, is isolated from the fermentation broth of the bacterium *Streptomyces cyanogriseus* [95]. The following four-step synthesis of moxidectin from nemadectin was disclosed in a 1991 patent [96]. The secondary alcohol hydroxy group on the hexahydrobenzofuran moiety was chemoselectively protected by a *p*-nitrobenzoate (PNB) ester (**194**). Then the other secondary alcohol hydroxy group was subjected to Albright–Goldman oxidation (DMSO/acetic anhydride), to give ketone **195**, which was converted to *O*-methyl oxime **196**, and the PNB group was removed by alkaline hydrolysis (without any damage to the macrolide ester linkage) to give moxidectin (Scheme 50).

## 20. Ubrogepant

**Figure 19:**
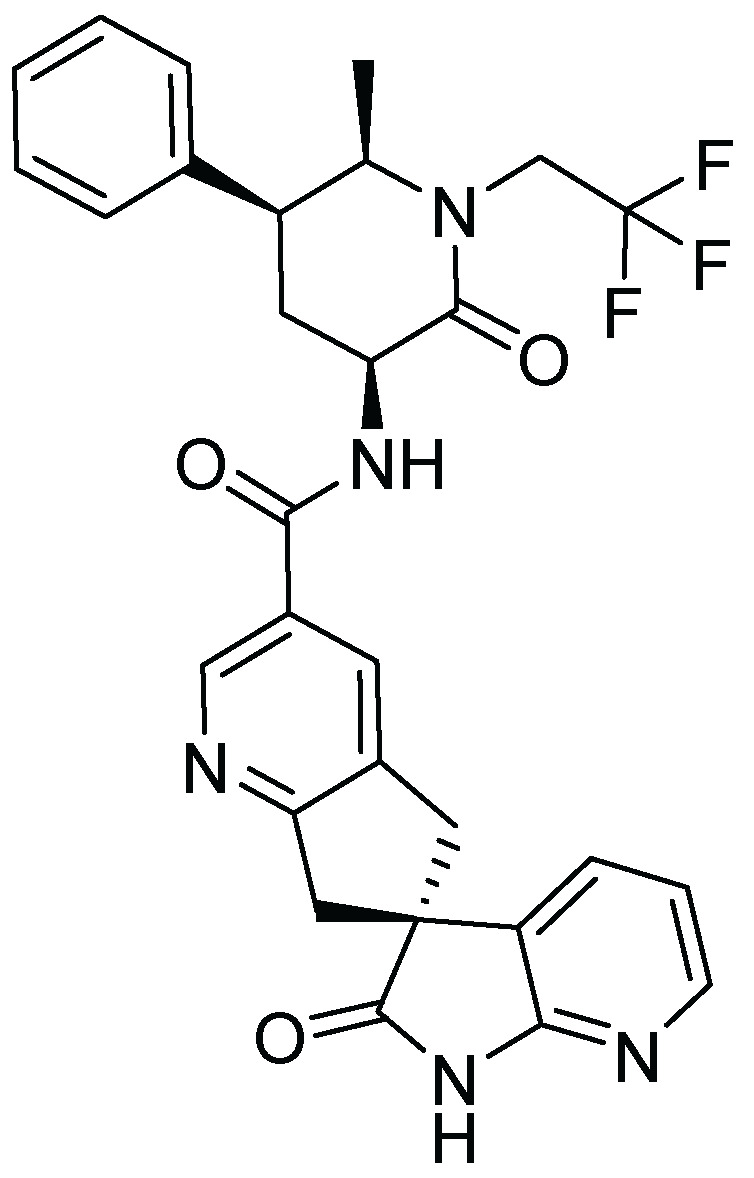
Structural formula of ubrogepant.

Migraine is a debilitating disease which was traditionally treated only symptomatically by serotonin receptor modulators or non-steroidal anti-inflammatory drugs. However, more recent in-depth investigation of the mechanisms underlying the onset of migraine established the calcitonin gene related peptide (CGRP) as a primary player in this pathological process. Produced in peripheral and central neurons, CGRP is a potent vasodilator and mediator of nociception. Thus, antagonizing the binding of CGRP to its receptor was established as a novel approach to the treatment of acute migraine at the fundamental level [97]. Ubrogepant (Figure 19) is the first-in-class drug developed by Merck and Co., and was approved in 2020 [98].

**Scheme 51:**
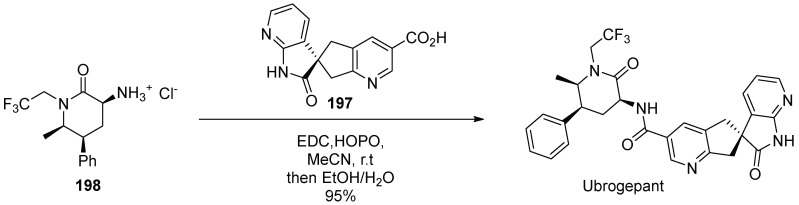
Final amidation in the assembly of ubrogepant.

The detailed process of the chemical route towards ubrogepant was published by the Merck team in 2017 [99]. The final step in the assembly of the target molecule was the amidation of the enantiopure spirocyclic carboxylic acid **197** with the enantiopure amine **198** hydrochloride salt (Scheme 51).

**Scheme 52:**
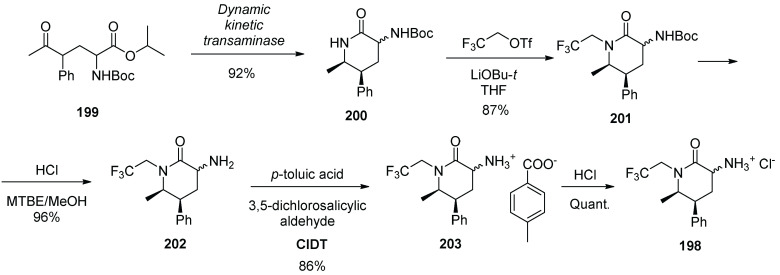
Merck synthesis of enantiopure 3-amino piperidone **198** [99].

Therefore, it is the assembly of enantiopure building blocks **197** and **198** which required most of the innovation. The synthesis of amine **198** started with a diastereomeric mixture of keto esters **199**. The latter was subjected to dynamic kinetic transaminase-mediated cyclization (using an evolved enzyme strain) to give piperidone **200** as a 3:2 mixture of epimers. *N*-Alkylation of the latter with 3,3,3-trifluoroethyl triflate afforded predominantly compound **201** (separated in 87% yield from the unreacted starting material and bis-alkylation product). Removal of the Boc group gave the epimeric amine **202**, which was subjected to crystallization-induced diastereoselective transformation (CIDT) with p-toluic acid and 3,5-dichlorosalicylic aldehyde to give building block **203**, which was converted to the hydrochloride salt **198** (Scheme 52).

**Scheme 53:**
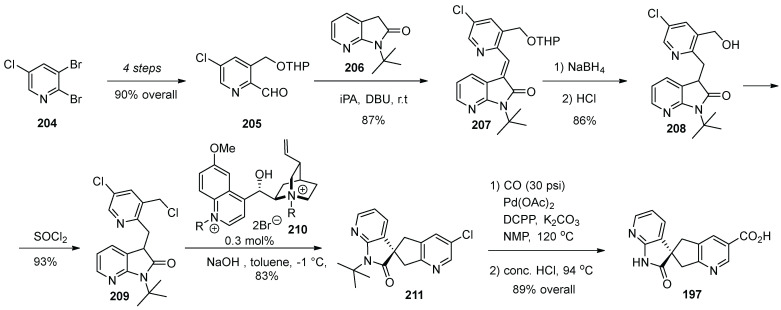
Merck synthesis of enantiopure spiro acid **197** [99].

The spirocyclic carboxylic acid **197** was assembled as follows. The 2,3-Dibromo-5-chloropyridine (**204**) was transformed in four steps (involving two additions of pyridine magnesium species to DMF) to hte tetrahydropyran-protected aldehyde **205**. The latter was condensed with 7-azaindolin-2-one **206** to give the olefin **207**, which was reduced by sodium borohydride, and the THP group was removed to give the alcohol **208**. Conversion of **208** into the alkyl chloride **209** set the scene for spirocyclization. On the action of aqueous sodium hydroxide, in the presence of the cinchona alkaloid-derived chiral catalyst **210**, chloride **209** underwent an efficient and enantioselective intramolecular *C*-alkylation to form the spirocycle **211**. Palladium-catalyzed carbonylation of the chloropyridine portion in the latter, followed by the removal of the *tert*-butyl group from the lactam nitrogen atom, afforded the key spirocyclic carboxylic acid **197** (Scheme 53).

**Scheme 54:**
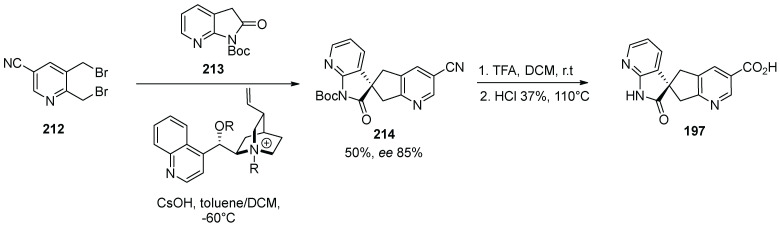
Phase-transfer-catalyzed asymmetric synthesis of spiro acid **197**.

An alternative approach to spirocyclic carboxylic acid **197** was reported in 2022 by Hu and co-workers [100]. It involves the direct asymmetric spiroannulation of Boc-protected 7-azaindolin-2-one (**213**) with the doubly benzylic bis-bromide **212**. Thus, from cheap and readily available starting materials, spirocycle **214** was obtained in good yield and enantioselectivity. Removal of the Boc group followed by nitrile hydrolysis gave the requisite spiro acid **197**, thus completing a formal synthesis of ubrogepant (Scheme 54).

## 21. Oliceridine

**Figure 20:**
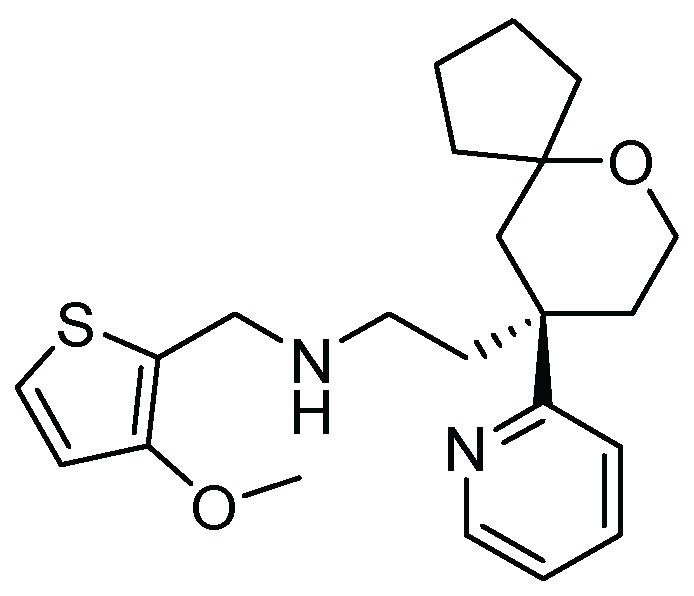
Structural formula of oliceridine.

Oliceridine (Figure 20), a spirocyclic biased [101] agonist of μ-opioid receptors was developed by Trevena, Inc. and was approved in 2020 for the intravenous treatment of acute moderate-to-severe post-operative pain [102].

**Scheme 55:**
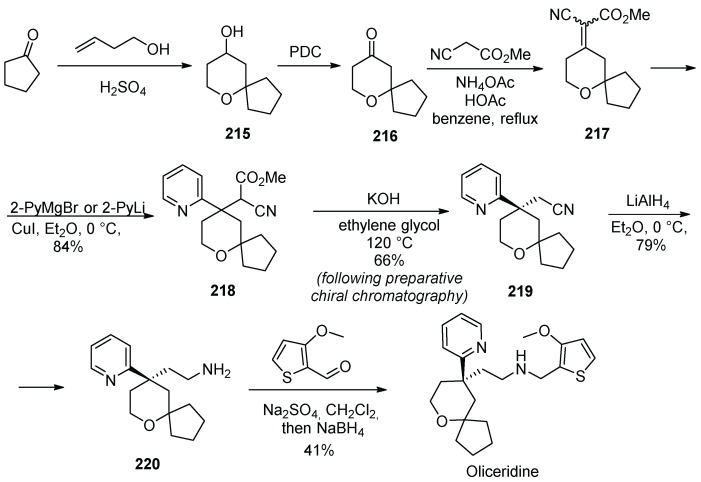
Synthesis of Oliceridine by Trevena, Inc [103].

The only full detailed route to the oliceridine drug substance is the original Trevena, Inc. approach, patented in 2012 [103]. The source of the spirocyclic motif was the ketone **216**, obtained by the oxidation of the perhydropyran-4-ol **215**, synthesized, in turn, by the Prins cyclization between cyclopentanone and homoallylic alcohol [104]. Base-promoted condensation with methyl cyanoacetate afforded the olefin **217**, to which pyrid-2-yl organometallic reagent (magnesium or lithium) was added in conjugate fashion, to give the cyano ester **218**. The latter was subjected to basic hydrolysis and decarboxylation at elevated temperature to afford the racemic product, which was resolved by preparative chiral supercritical fluid chromatography to give the enantiopure nitrile **219**. This product was reduced using LAH and the resulting amine **220** was reductively alkylated with 3-methoxythiophene-2-carboxaldehyde to give oliceridine (Scheme 55).

## 22. Risdiplam

**Figure 21:**
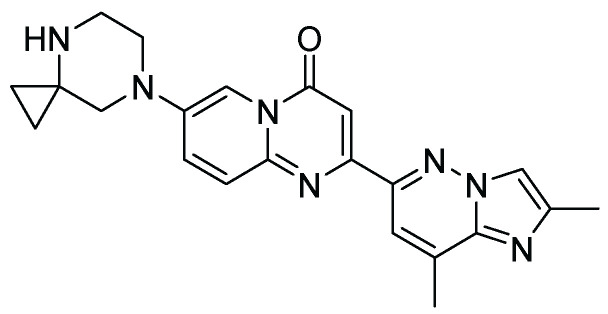
Structural formula of risdiplam.

Risdiplam (Figure 21) is the first oral medication to treat spinal muscular atrophy, an orphan disease [105]. The drug was developed by PTC Therapeutics and the Spinal Muscular Atrophy Foundation, and received FDA approval in 2020 for the treatment of the disease in patients aged 2 months or older [106]. The drug acts by modifying mRNA splicing by binding to the survival of motor neuron 2 (SMN2) [107]. It is currently marketed by Genentech, a subsidiary of Roche.

The synthetic routes to risdiplam disclosed in the original chemical process patents by Roche rely on the commercial supply of the spirocylic piperazine building block, and thus do not involve the formation of the spirocyclic moiety in the course of the synthesis.

**Scheme 56:**
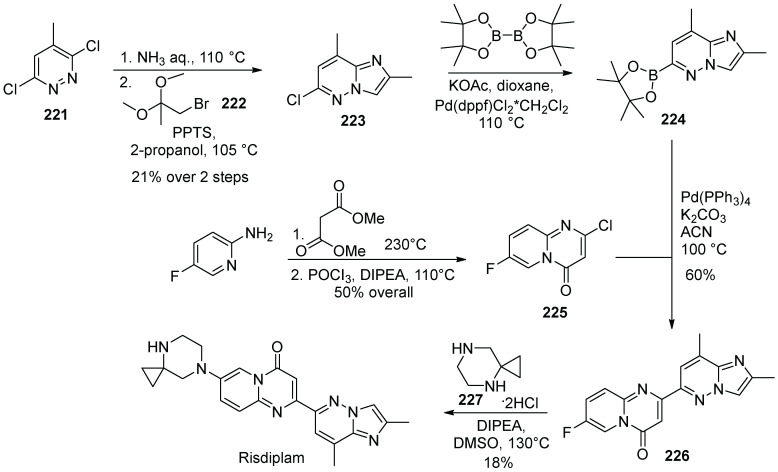
A 2015 synthesis of risdiplam by Roche [108].

In their 2015 patented route, PTC Therapeutics and Roche scientists started with 3,6-dichloro-5-methylpyridazine (**221**), in which the less accessible chlorine atom was substituted with ammonia and the imidazo ring was formed on condensation with bromoacetone dimethyl ketal (**222**). The overall yield of the resulting imidazo pyridazine **223** was obtained in relatively low yield (unsurprisingly, considering the unfavorable course of the initial nucleophilic aromatic substitution). Compound **223** was coupled, under palladium(II) catalysis, with bis(pinacolato)diboron, to give boronic acid **224**. The latter was coupled again, in Suzuki fashion, with the pyrido pyrimidone building block **225** (obtained, in turn, from 2-amino-5-fluoropyridine on condensation with dimethyl malonate). The Susuki coupling **224** + **225** resulted in the core risdiplam scaffold **226**, which was decorated with spirocyclic piperazine **227** under thermal conditions, to give risdiplam (Scheme 56) [108].

**Scheme 57:**
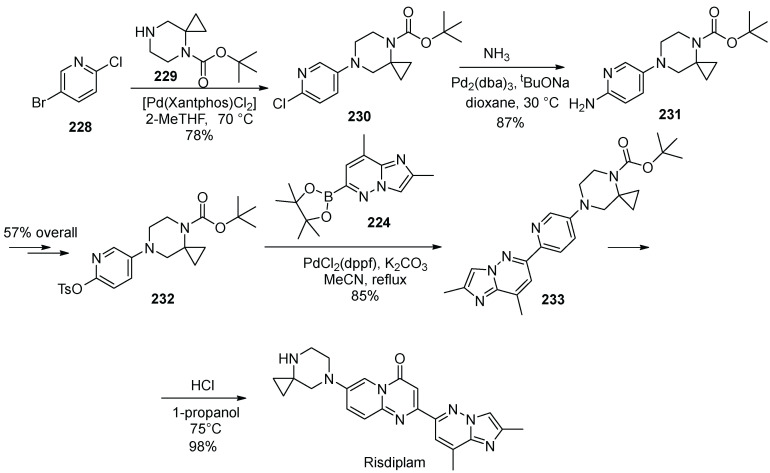
A 2019 synthesis of risdiplam by Roche [109].

For the more recent route following their 2019 patent application, Roche scientists started by involving 5-bromo-2-chloropyridine (**228**) in Buchwald–Hartwig amination with Boc-protected spirocyclic piperazine (**229**). The unreacted, under Pd(II)-catalyzed conditions, chlorine atom in **230** was displaced by ammonia under Pd(0) catalysis and the amino portion of the resulting intermediate 231 was converted in two steps into tosylate **232**. The latter was coupled with boronic acid **224** (prepared as described in the previous Roche patent) in Suzuki fashion, and the Boc group in the resulting coupling product **233** was removed to give risdiplam (Scheme 57) [109].

## 23. Atogepant

**Figure 22:**
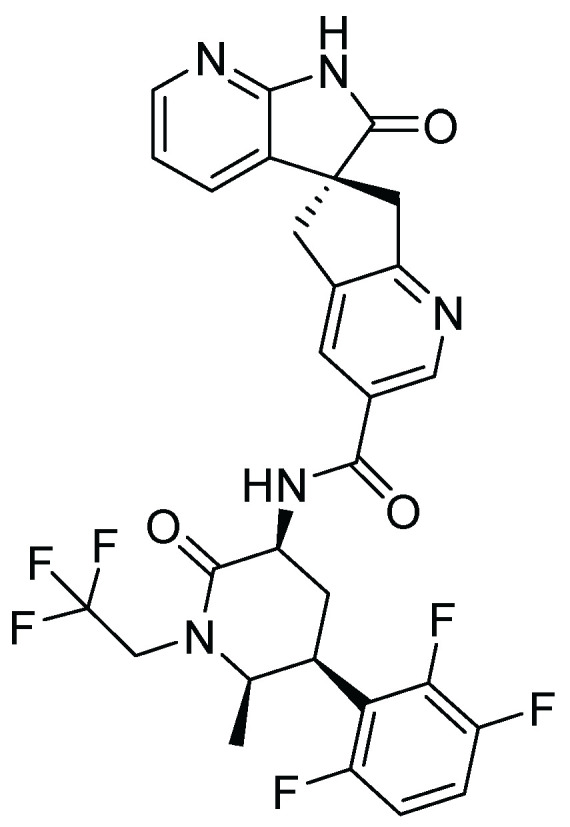
Structural formula of atogepant.

Atogepant (Figure 22) is a next-in-class antagonist of the calcitonin gene- related peptide (CGRP) is a direct analog of ubrogepant (the first-in-class CGRP antagonist developed by Merck, vide supra), which is different from ubrogepant since it contains a 2,3,6-trifluorophenyl ring. Discovered by Merck and developed by AbbVie, atogepant was approved in 2021 for the preventive treatment of episodic migraine in adults [110].

**Scheme 58:**
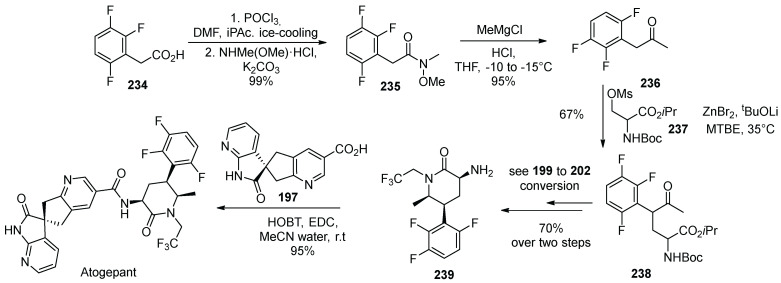
Synthesis of atogepant by Merck [111].

The synthesis of atogepant was described in detail in a 2016 patent application by Merck [111], and it relies on essentially the same routine as was used in the preparation of ubrogepant. Specifically, the same enantiopure spiro acid **197** was in use. The enantiopure 3-aminopyrid-2-one building block was also assembled in a fashion similar to the amine half of ubrogepant (**202**). The synthesis commenced with the phenylacetic acid, which was converted to the respective acyl chloride and then the Weinreb amide **235**. The latter was treated with methylmagnesium chloride to give the methyl ketone **236**. The latter was alkylated at the most acidic, benzylic position with mesylate **237** to give keto ester **238**. The latter is a direct analog of compound **199** as a precursor to **202**—and the exact same routine, involving the use of dynamic kinetic transaminase, was used to convert compound **238** to enantiopure 3-aminopyrid-2-one **239**. Amidation of the spiro acid **197** with the amine **239** gave atogepant (Scheme 58).

## 24. Trilaciclib

**Figure 23:**
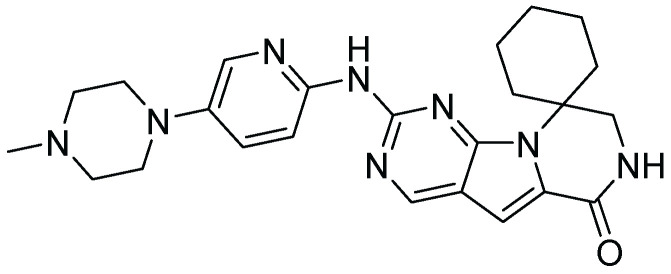
Structural formula of trilaciclib.

Trilaciclib (Figure 23) spirocyclic inhibitor of cyclin-dependent kinases 4 and 6 (CDK4 and CDK6) was developed by G1 Therapeutics. The drug was indicated for cancer patients receiving certain types of chemotherapy to protect the bone marrow from chemotherapy-induced damage. As trilaciclib was the first-in-class drug developed for this important indication, it received a priority review and a breakthrough therapy designation from the FDA. It was approved for medical use in 2021 [112].

The drug’s originator patented two synthetic routes to the compound, both involving an intramolecular *N*-acylation as the key step in the construction of the spirocyclic moiety [113,114]. Of these two approaches, the later one [114] is described in much greater detail, and is therefore the API production route which we chose to discuss herein.

**Scheme 59:**
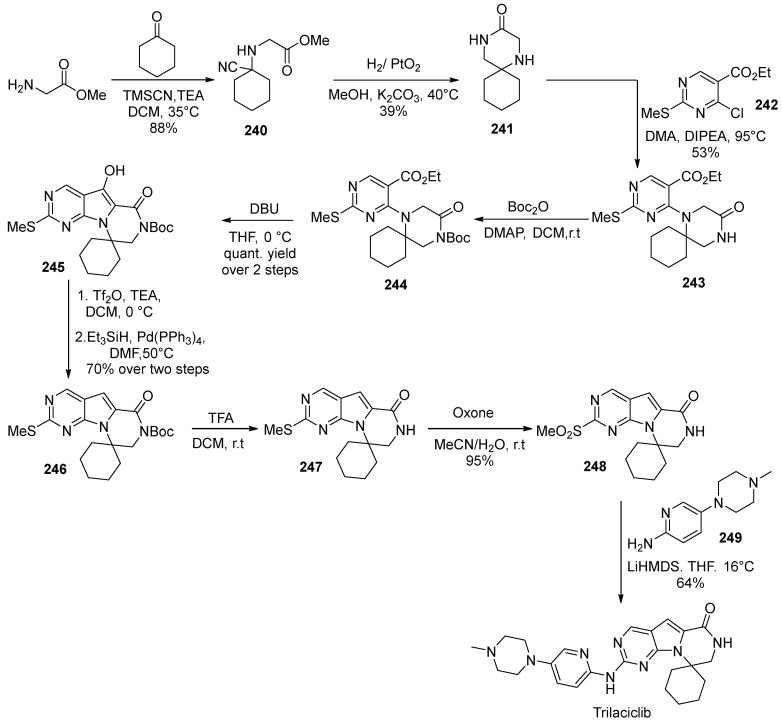
G1 Therapeutics industrial synthesis of trilaciclib [114].

The synthesis commences with an efficient and high-yielding Strecker reaction of cyclohexanone with glycine methyl ester and trimethylsilyl cyanide, to afford the amino nitrile **240**. The latter was hydrogenated over platinum(IV) oxide. This caused the reduction of the nitrile to the aminomethyl compound, which underwent lactamization to afford the key spirocyclic building block **241**. Without protection, the sterically hindered secondary amine **241** was used to displace the labile chlorine atom in the pyrimidine template **242**. The nucleophilic aromatic substitution reaction proceeded under forcing conditions and afforded the spirocycle **243**, which was now set for the formation of the pyrrolo portion of the target molecule. As this step would require C-H deprotonation of the piperazinone moiety, the lactam nitrogen of intermediate **243** was Boc-protected to give compound **244**. Treatment of the latter with DBU at 0 ℃ in THF triggered the desired deprotonation and intramolecular cyclization, and gave the 3-hydroxypyrrolo compound **245** in quantitative yield over the last two steps (including the preceding Boc-protection). Clearly, the hydroxy group in **245** had to be removed, which was achieved by *O*-triflation of the compound with the subsequent palladium(0)-catalyzed reductive coupling with triethylsilane. The two steps resulted in 70% combined yield and afforded the spirocyclic pyrrolo pyrimidine **246**, which was deprotected with trifluoroacetic acid to give the advanced trilaciclib precursor **247**. To complete the synthesis, the methylthio group in **247** was oxidized to the better, leaving methylsulfonyl group with oxone in aqueous acetonitrile, to give **248** in excellent yield. The final nucleophilic aromatic substitution step between the spirocyclic template **248** and the pyridine amine **249** (prepared, in turn via nucleophilic aromatic substitution with 5-halopyridine precursor and *N*-methylpiperazine) required the use of a strong base (LiHMDS), and gave trilaciclib (Scheme 59).

## 25. Conclusions and Perspectives

As noted previously, the use of spirocycles in drug discovery and medicinal chemistry has seen an explosive rise [2]. This trend is clearly reflected in the number of drugs containing a spirocyclic motif which received approval for medical use over the years. Indeed, over 50% of such drug molecules have been approved in this century. A range of synthetic methods to form a spirocyclic scaffold have been employed in the production routes towards these drugs, although at times the source of the spirocyclic moiety is the naturally occurring, biosynthesized compound or a commercially available spirocyclic building block (Table 1).

The trend is likely to continue, resulting in new advanced drug candidates and approved drug molecules, which mandates that the arsenal of synthetic methods applicable to spirocycle formation is expanded beyond the most popular key steps such as lactonization and lactamization, cyclocondensation (including ketalization and acetalization) and including intramolecular *C*-alkylation and acylation. Given the rate of development of synthetic approaches towards the construction of spirocyclic molecules employing various modern techniques (e.g., photochemistry, multicomponent reactions, diazo chemistry, asymmetric catalysis, flow synthesis, etc.) which is reflected in the high annual publication activity, we have no doubt that many of them will find application in the field of both drug design and industrial production in the near future.

## Figures and Tables

**Table 1:** Summary of approved spirocyclic drugs: year of approval and origin of spirocyclic moiety.

Entry	Drug Name	Year of Approval	Origin of Spirocycle
1	Griseofulvin	1959	Cycloaddition, intramolecular Claisen condensation
2	Spironolactone	1960	Lactonization
3	Fluspirilene	1970	Cyclocondensation
4	Fenspiride	1970	Lactamization
5	Amcinonide	1979	Ketalization
6	Guanadrel	1979	Ketalization
7	Buspirone	1986	Commercially available spiro building block
8	Ivermectin	1986	Biosynthesis
9	Rifabutin	1992	Cyclocondensation
10	Spirapril	1995	Thioketalization
11	Irbesartan	1997	Cyclocondensation
12	Cevimeline	2000	Acetalization
13	Eplerenone	2002	Biosynthesis
14	Drospirenone	2000	Lactonization
15	Ledipasvir	2014	Cyclopropanation
16	Rolapitant	2015	Lactamization, ring-closing metathesis
17	Apalutamide	2018	Lactonization
18	Moxidectin	2018	Biosynthesis
19	Ubrogepant	2020	Intramolecular *C*-alkylation
20	Oliceridine	2020	Prins reaction
21	Risdiplam	2020	Commercially available spiro building block
22	Atogepant	2021	Intramolecular *C*-alkylation
23	Trilaciclib	2021	Intramolecular Claisen condensation

## Data Availability

Not applicable.
